# Seed Halopriming as an Effective Strategy to Enhance Salt Tolerance in *Cakile maritima*: Activation of Antioxidant and Genetic Responses

**DOI:** 10.3390/antiox14030353

**Published:** 2025-03-18

**Authors:** Roser Tolrà, Carlos González-Cobo, Isabel Corrales, Rosa Padilla, Mercè Llugany

**Affiliations:** Plant Physiology Group (BABVE), Universitat Autònoma de Barcelona, 08193 Barcelona, Spain; carlos.gonzalezc@uab.cat (C.G.-C.); isabel.corrales@uab.cat (I.C.); rosa.padilla@uab.cat (R.P.)

**Keywords:** salinity, *Cakile maritima*, seed priming, antioxidant response, *SOS1*, *SOS2*, *NHX1*, *WRKY25*, salt tolerance, oxidative stress

## Abstract

Global food demand and insecurity are intensifying due to rapid population growth, the loss of arable land, climate change, and pollution. Among the critical challenges in global agriculture is soil salinization, in which high NaCl concentrations can severely inhibit germination and crop establishment. *Cakile maritima*, a halophyte from the *Brassica* genus, can tolerate salinity levels up to 400 mM NaCl, far exceeding the tolerance of most crops, making it a promising model for studying salt stress resistance. This study investigates the effects of seed halopriming as an effective strategy to enhance salt tolerance in *C. maritima*. The research evaluates germination rates, seedling establishment, mineral status, oxidative stress markers, and genetic responses under increasing NaCl concentrations. Halopriming with NaCl pre-activates the plant’s antioxidant defence mechanisms and upregulates stress-responsive genes, improving the plant’s resilience to saline conditions. While salinity caused significant physiological challenges, primed seeds demonstrated superior performance compared to non-primed controls, with enhanced germination and an improved tolerance to oxidative stress. These findings underscore the potential of halopriming as a cost-effective and sustainable technique to improve crop performance in saline environments. This study highlights the importance of advancing seed priming technologies for developing resilient crops to address global food security challenges in the face of climate change.

## 1. Introduction

The growing global demand for food, exacerbated by climate change (CC) and environmental pollution, has heightened the need for innovative strategies to enhance agricultural productivity and ensure food security. Rapid population growth and the continuous loss of arable land are intensifying concerns about food insecurity. Climate change is reshaping traditional cultivation patterns, significantly impacting crop phenology, reproduction, and growth. For instance, projections estimate a loss of approximately 280 million tons of cereal production in Asia and Africa by the end of this century due to CC [1]. These changes also affect seed viability, altering key traits such as size, dormancy, productivity, and quality [2].

Among the major challenges in agriculture, soil salinization presents a critical barrier to crop establishment, threatening yields and global economic stability. High salinity, particularly NaCl concentrations between 300 and 400 mM, can completely inhibit germination in most species [3]. The United Nations Food and Agriculture Organization (FAO) estimates that over one billion hectares of soil is affected by excessive salinity or sodium ions [4]. Efforts to address salinity have focused on enhancing plant tolerance through genetic selection and biotechnological interventions. However, the genetic potential of seeds remains a limiting factor, directly influencing germination and crop productivity. In natural environments, seeds encounter complex microenvironmental conditions—including pH, soil texture, moisture, temperature, light, and oxygen levels—that significantly influence their germination and development, as these factors are often “imprinted” on the seeds [5].

**Figure 1:**
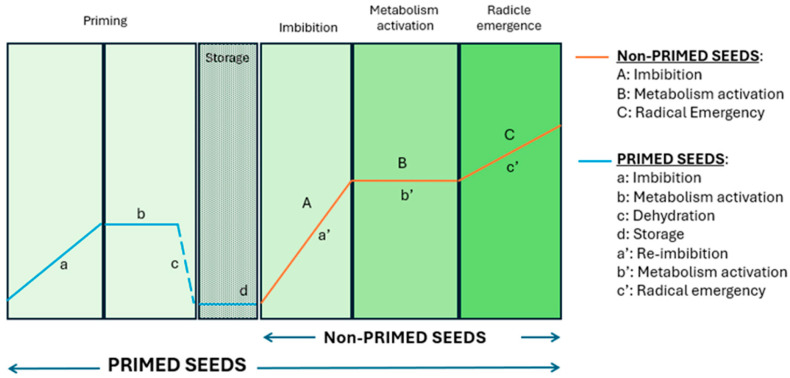
Illustrative graph depicting the phases of germination in non-primed and primed seeds (designed by the authors).

Seed priming has emerged as a promising, cost-effective strategy to improve seed performance and resilience to abiotic stresses like salinity and drought. This pre-sowing technique involves the controlled hydration of seeds in specific solutions, activating pre-germinative metabolic processes but maintaining the seeds’ moisture content below the threshold required for triggering radicle emergence (Figure 1), and subsequently drying the seeds for storage [6,7,8].

Priming imparts a “memory” of the specific stress the seed has encountered, enabling seedlings to experience significantly reduced effects upon subsequent exposure to the same stress factor and allowing for a much faster response [9]. Seed priming enhances germination rates, reduces germination time, and boosts seedling vigour while increasing plant resilience to biotic and abiotic stress factors [10]. This practice also promotes synchronized germination, better seedling establishment, and improved yields under stressful conditions, owing to mechanisms like DNA repair activation and antioxidant responses, but also under normal conditions [11,12,13].

Different priming methods—such as hydropriming, halopriming, osmopriming, and nutripriming—are used to address specific stress factors. Among these, NaCl-based halopriming has demonstrated efficacy in enhancing salt tolerance, as exposure to salinity primes seeds for faster and more robust responses upon subsequent stress encounters [14,15]. However, the effectiveness of seed priming varies with species and stress conditions, necessitating tailored approaches for different crops and environments.

Many modern crops are glycophytes, which are sensitive to salt stress. In contrast, halophytes—salt-tolerant plants—can endure salinity levels exceeding 200 mM, providing valuable insights into the mechanisms of salinity tolerance [16]. These plants possess specialized physiological and morphological adaptations, such as ion homeostasis, osmotic regulation, and reactive oxygen species (ROS) detoxification, which enable them to thrive under extreme conditions [17,18,19].

*Cakile maritima*, commonly known as sea rocket, is a highly salt-tolerant halophyte and a member of the Brassicaceae family, which includes economically important crops like mustard and rapeseed. Its ability to thrive in salinity levels up to 400 mM NaCl, far exceeding the tolerance threshold of most crops, makes it an excellent model for studying salt tolerance mechanisms which can provide strategies for improving stress resilience in related crop species [20]. Despite its genetic similarity to *Arabidopsis thaliana*, *C. maritima* exhibits superior antioxidant capacity, phenolic compound production, and efficient Na^+^ transport, which collectively mitigate salt stress [21]. This enhanced antioxidant capacity, together with its edible leaves and stems, has made *C. maritima* a candidate explored in cuisine innovation [22]. Additionally, it can be used for phytoremediation purposes or to restock areas without vegetation due to its high salinity content [23].

In this study, we explore the potential of halopriming using NaCl to enhance seed performance and plant resilience to salinity. By leveraging the inherent salt tolerance of *C. maritima*, we examine its physiological responses to varying salinity levels, focusing on both pre- and post-priming effects. Our objective was to assess the effects of salt stress after 7 days of exposure, capturing the physiological and biochemical adjustments in the later stages of stress response instead of early responses that are focused on initial cellular signalling and stress perception. This research aims to evaluate the feasibility of using halopriming as a sustainable, eco-friendly approach to developing crops better equipped to withstand salinity, addressing the urgent challenges posed by climate change and soil degradation.

## 2. Materials and Methods

### 2.1. Plant Material

Seeds of *Cakile maritima* were purchased from CANTUESO Natural Seeds (Córdoba, Spain). The seeds were manually stratified and surface-sterilized by soaking them in 70% ethanol (*v*/*v*) for 1 min, followed by immersion in 30% (*v*/*v*) commercial Clorox bleach with a drop of Tween-20 for 5 min. After sterilization, the seeds were rinsed five times with sterile 18 MΩ Milli-Q water.

Seeds were primed by hydrating them in ultrapure water (18.2 MΩ·cm) containing different NaCl concentrations: water (WPr), 200 mM NaCl (200Pr), and 400 mM NaCl (400Pr). The priming process lasted 12 h in the dark. After priming, the seeds were dried and stored in the dark at room temperature for 48 h.

### 2.2. Experimental Design

A germination assay was conducted using 20 seeds from each priming group (WPr, 200Pr, 400Pr) and 20 non-primed seeds (NPr). All seeds were sown on plates containing various NaCl treatments: control (CK, 0 mM NaCl), 200 mM NaCl, and 400 mM NaCl. The plates were incubated in the dark at 22 °C for 5 days. Seeds were considered germinated when the emerging radicle was visible. Three independent replicates were performed, and the germination percentage (GP) was calculated using the following formula:

$$GP=\left(\frac{Total\;germinated\;seeds}{Total\;number\;seeds}\right)\times 100$$

In a second experiment, physiological, biochemical, and genetic parameters were analysed in 4-week-old *C. maritima* plants grown from the previous primed and non-primed seeds under varying NaCl concentrations. Seeds were sown in pots containing a 1:1 sand-perlite mixture and watered with distilled water for 1 week until germination. After germination, seedlings were grown for 3 weeks and irrigated every 2 days with ½ strength Hoagland nutrient solution. At the 4-week mark, plants were exposed to the same salt concentrations used in the germination assay (CK, 200 mM NaCl, and 400 mM NaCl) for 1 week. The plant material was then harvested, frozen in liquid nitrogen, and stored at –80 °C for further analysis.

### 2.3. Biomass Production and Relative Water Content

Fresh weight (FW) and dry weight (DW) were recorded at the end of the assay, and the relative water content (RWC%) was calculated using the following formula:

$$RWC=\left(\frac{FW-DW}{FW}\right)\times 100$$

### 2.4. Photochemical Efficiency and Stomatal Density

Just before harvesting, the photochemical activity and efficiency of PSII (Fv/Fm) were measured using chlorophyll fluorescence with a MINI-PAM-II system (Heinz Walz GmbH, Effeltrich, Germany). Measurements were performed on randomly selected, fully expanded leaves that had been dark-adapted for 30 min (blue LED: 470 nm; standard PAR measurement: 0.05 μmol m^−2^ s^−1^). Readings were taken at wavelengths above 630 nm, both before and after salt treatment, on two visually healthy leaves from three plants for each salinity treatment.

Stomatal density (SD) was determined in 3 basal leaves and 3 mid-height leaves for each priming condition and treatment. To obtain the samples, a layer of fixing lacquer was placed on the basal face of the leaves and allowed to dry for about 5 min. Once dry, the lacquer was separated with the help of tweezers, placed on a slide, and covered with a coverslip. The preparations were visualized under a microscope (10×) (Leica Zoom 2000. Buffalo, NY, USA), and the field area and stomata count were calculated in two different fields, avoiding the leaf edge and areas close to the central rib. The stomatal density was calculated as the number of stomata per mm^2^, using the WPr/CK combination as the reference (control) and assigning it a baseline value of 100%. This allowed for the relative comparison of stomatal densities across different treatments.

### 2.5. Determination of Mineral Elements 

The concentrations of sodium (Na), potassium (K), phosphorus (P), magnesium (Mg), calcium (Ca), sulphur (S), zinc (Zn), iron (Fe), manganese (Mn), copper (Cu), boron (B), and molybdenum (Mo) were analysed in both shoots and roots using inductively coupled plasma optical emission spectroscopy (ICP-OES; Thermo Jarrell-Ash, Model 61E Polyscan, UK). Dry plant material (0.1 g) was acid-digested in Pyrex tubes using concentrated HNO_3_ at 110 °C for 5 h. The digestion process was carried out in a hot block digestion system (SC154-54-Well Hot Block™, Environmental Express, Charleston, SC, USA) following the method of Soltanpour et al. (1977) [24]. Values are presented in radial plots of normalized mineral nutrient differences in which axes display Z-scores calculated per element.

### 2.6. Oxidative Stress Markers

The level of lipid peroxidation was estimated by determining the concentration of malondialdehyde (MDA) following the method of Draper et al. (1993) [25]. Approximately 0.5 g of fresh leaf tissue was homogenized in 0.1% (*w*/*v*) trichloroacetic acid (TCA) and centrifuged at 12,000 rpm for 15 min. For MDA determination, 0.5 mL of the resulting supernatant was mixed with 0.5 mL of 0.5% (*w*/*v*) thiobarbituric acid (TBA) in 20% (*w*/*v*) TCA. The mixture was incubated at 95 °C for 25 min and then rapidly cooled in an ice bath. After cooling, the mixture was centrifuged at 8000 rpm for 6 min. The absorbance of the supernatant was measured at 532 nm using an MDA extinction coefficient of 155 mM cm^−1^, and results are expressed as µmol MDA g^−1^ FW.

Hydrogen peroxide (H_2_O_2_) levels were quantified following the method described by Velikova et al. (2000) [26]. Fresh leaf tissue was homogenized in 0.5% (*w*/*v*) trichloroacetic acid (TCA) and centrifuged at 12,000 rpm for 15 min. For H_2_O_2_ determination, an aliquot of the supernatant was mixed with 100 mM phosphate buffer (pH 7.0) and 1 M potassium iodide (KI). The reaction mixture was incubated in the dark for 1 h, and the absorbance was measured at 390 nm. A standard curve of known H_2_O_2_ concentrations was used for quantification, and results are expressed as mmol H_2_O_2_ g^−1^ FW.

### 2.7. Total Antioxidant Capacity and Antioxidant Enzyme Activity

The total antioxidant capacity (TEAC) was analysed using the 2,2-Diphenyl-1-picrylhydrazyl (DPPH) method described by Brand-Williams et al. (1995) [27]. Briefly, 0.5 g FW of plant material was extracted with 80% methanol to obtain a final volume of 10 mL of methanolic extract. The extract was stored at −20 °C until analysis. For TEAC determination, the DPPH solution was prepared by dissolving DPPH in 0.1 mM iminoazanium in methanol. Each sample or Trolox standard (0.1 mL of methanolic extract) was mixed with the DPPH solution. After a 30 min reaction, absorbance was measured at 515 nm using a plate reader spectrophotometer (TECAN, Männedorf, Switzerland). The antioxidant capacity was expressed as µmol Trolox equivalent per gram of FW (µmol Trolox g^−1^ FW).

The preparation of extracts for determining catalase (CAT) and superoxide dismutase (SOD) activities followed the method of Alexieva et al. (2001) [28]. Frozen plant tissue (0.1 g) was homogenized in 50 mM potassium phosphate buffer (pH 7.4) containing 0.5% (*v*/*v*) Triton X-100, 1 mM EDTA, and polyvinylpyrrolidone (PVP) in a precooled mortar. The homogenate was centrifuged at 10,000× *g* for 15 min at 4 °C, and the supernatant was used for enzymatic activity assays. Protein concentration in the extracts was quantified using Bradford reagent.

SOD activity was determined using the method of Beauchamp and Fridovich (1973) [29]. In this assay, superoxide anions generated by slightly excited riboflavin reduce nitroblue tetrazolium (NBT), forming a blue formazan product measured at 560 nm. The reaction mixture contained 0.02 mg mL^−1^ of protein extract, 75 µM NBT, 777 µM methionine, 0.54 µM EDTA, and 8 µM riboflavin in final volume of 1 mL. Absorbance was measured at 560 nm, first in the dark and then after 7 min of UV light exposure. One unit of SOD activity was defined as the amount of enzyme required to inhibit in a 50% reduction in NBT.

CAT activity was measured according to the protocol of Aebi (1984) [30]. The assay mixture consisted of 980 µL of potassium phosphate buffer (pH 7.4), 10 µL of 5 mM H_2_O_2_, and 10 µL of plant extract. CAT activity is defined as the amount of enzyme that decomposes 1 mmol of H_2_O_2_ per minute, measured by a decrease in absorbance at 240 nm.

### 2.8. RT-qPCR Analysis of Genes Involved in Ion Homeostasis and Antioxidant Defence

Total RNA was extracted from leaves of plants grown from non-primed and primed seeds using the Maxwell RSC Plant RNA Kit (Promega Biotech Ibérica SL, Alcobendas, Spain) following the manufacturer’s protocol. RNA concentration was quantified using a Nanodrop spectrophotometer (Thermo Fisher Scientific, Barcelona, Spain). First-strand cDNA synthesis was performed using the iScript cDNA Synthesis Kit (Bio-Rad, Barcelona, Spain) according to the manufacturer’s instructions.

The target genes *CmSOS1*, *CmSOS2*, *CmHKT1*, and *CmWRKY25* were identified from Phytozome *C. maritima* assembly [31] based on their *Arabidopsis thaliana* orthologs using BLASTn, and primers were designed using Primer-BLAST platform (NIH, Rockville Pike, Bethesda, MD, USA) [32]. A list of primers is provided in Table 1.

Gene expression was analysed by RT-qPCR using the iTaq Universal SYBR Green Supermix kit (Bio-Rad). *CmUBQ10* expression was used as an internal reference gene. Relative gene expression was calculated using the 2^−ΔΔCt^ method by Livak et al. (2001) [33]. Three biological replicates and three technical replicates were included for each salt and priming condition to ensure reliability.

### 2.9. Statistical Analysis

One-way ANOVA and Tukey post hoc test were performed using JMP Pro 13 software (JMP Statistical Discovery, Cary, NC, USA) to determine the effect of the treatment for seed priming conditions. The statistical differences were referred to as significant when *p*-value < 0.05 (n = 6).

## 3. Results

### 3.1. Effect of Seed Priming on Physiological Parameters

**Figure 2:**
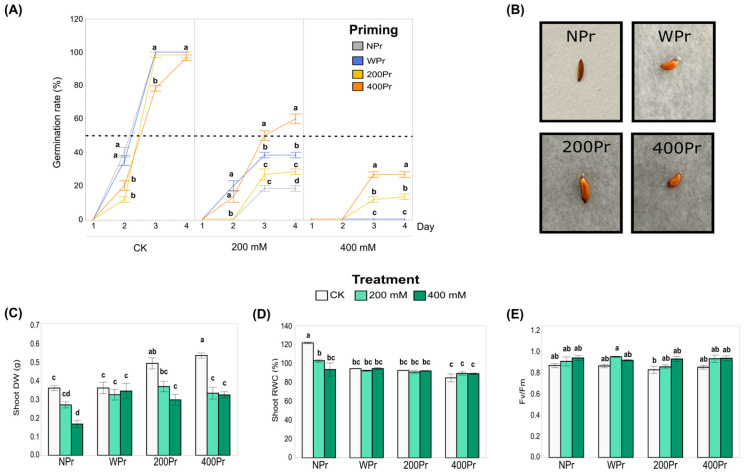
Influence of priming on the percentage of *C. maritima* seed germination under different NaCl conditions over 4 days. The dotted line indicates the 50% germination rate (**A**), Pictures of seeds from different priming groups (**B**), Biomass production expressed as dry weight (**C**), Relative water content (**D**), and Photosynthetic efficiency (**E**) in shoots of *C. maritima* plants grown from non-primed (NPr) and primed seeds (WPr, 200Pr, 400Pr) under different NaCl treatments (CK, 200 mM, and 400 mM NaCl) for 7 days. Values are presented as the mean ± SE (n = 6), except for the germination (n = 3; 20 seeds per replicate). Different letters indicate significant differences between treatments and priming groups, except in (**A**), in which they indicate differences only between priming groups per day (Tukey test, *p* < 0.05).

The germination percentage of both NPr and Pr seeds remained around 100% under the control conditions but declined significantly with increasing salt concentrations (Figure 2A). In all priming groups, germination began on day 2, and from day 4 onwards, it did not increase further. For the 200Pr and 400Pr groups, the number of germinated seeds on day 2 were significantly lower than in the NPr and WPr groups. Under the 200 mM NaCl treatment, the 400Pr group showed a greater germination rate compared to the rest of the priming conditions, being the only group for which the germination rate exceeded 50%. When the seeds were exposed to 400 mM NaCl, only the haloprimed ones were able to germinate, reaching germination rates of 13% and 27%, corresponding to the 200Pr and 400Pr groups, respectively. Seed colour, size, shape, and morphology did not change after the priming treatments (Figure 2B).

The shoot biomass of *C. maritima* was significantly affected by both seed priming and the salt treatment (Figure 2C). Under the control conditions (CK), plants grown from haloprimed seeds (200Pr and 400Pr) showed a significantly higher shoot dry weight compared to those grown from WPr and NPr seeds. Under the 200 mM NaCl treatment, no significant differences were observed among the priming groups. However, under the 400 mM NaCl treatment, the plants from the NPr seeds exhibited the lowest shoot biomass compared to all the other priming groups. These results suggest that seed priming can enhance the resilience of *C. maritima* to salt stress, although the effects vary depending on the type of priming and salt concentration.

In general, salinity causes a decrease in water content throughout the plant. In this study, plants grown from NPr seeds showed a significant reduction in the relative water content (RWC) in shoots when exposed to both 200 mM and 400 mM NaCl, compared to other priming groups (Figure 2D). No significant differences in RWC were observed among the priming groups under salt treatments, though a slight, non-significant reduction in RWC was noted in the primed plants compared to the control plants. Despite this, NaCl priming appeared to assist in the better maintenance of tissue hydration in the aerial parts of the plant, although the difference was not statistically significant. These results suggest that priming may help mitigate some of the negative effects of salt on plant hydration.

The Fv/Fm ratio, which serves as a key indicator of the plant’s photosynthetic capacity, remained largely unaffected across all priming groups, from the control to the highest salt concentration (Figure 2E). The only exception was the 200 mM NaCl treatment in the WPr seeds, which exhibited a significantly higher value than 200Pr under the control conditions. Most Fv/Fm values remained close to 0.8, indicating that the plants maintained good physiological condition. Consequently, neither seed priming nor salt treatment appeared to have a significant effect on the photosynthetic performance of *C. maritima*.

The results from Table 2 show that seed priming with NaCl influences stomatal density (SD) in both basal and mid-height leaves of *C. maritima* plants under varying salt treatments. In basal leaves, the WPr seeds exhibited an increase in SD under 400 mM NaCl (110.5%), while the 200Pr seeds showed a significant decrease (75.2%). The highest SD is shown in the 400Pr seeds at 400 mM NaCl (131.0%), indicating better salt tolerance. In mid-height leaves, the WPr seeds showed a significant reduction in SD under both 200 mM NaCl (63.0%) and 400 mM NaCl (66.6%). The 200Pr seeds also exhibited a decrease in SD with higher salt concentrations, but the reduction was less pronounced than in the WPr seeds. Interestingly, the 400Pr seeds maintained a relatively high SD under a high salinity, with values reaching 82.6% at 400 mM NaCl, suggesting that NaCl priming might enhance stomatal maintenance under salt stress. Overall, the results suggest that NaCl priming, particularly at higher concentrations (400Pr), may help *C. maritima* better manage stomatal density under salt stress, especially in basal leaves.

### 3.2. Effect of Seed Priming on Na^+^ and K^+^ Accumulation and Mineral Nutrition

**Figure 3:**
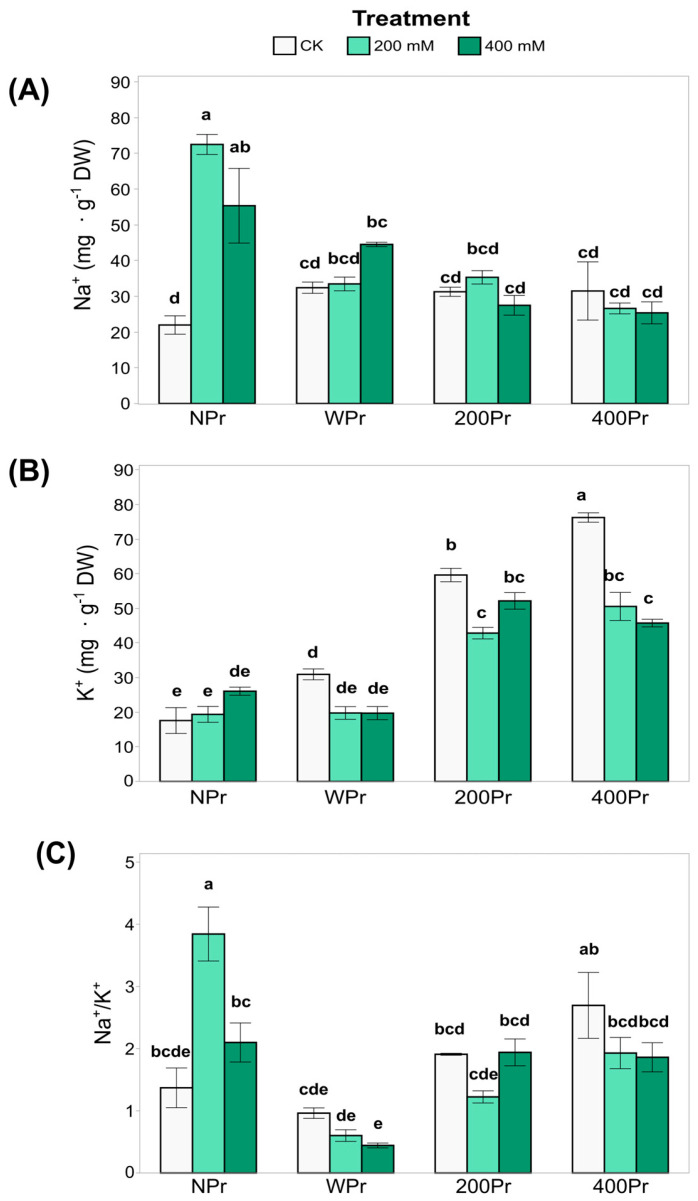
Concentration of Na^+^ (**A**) and K^+^ (**B**), and the ratio Na^+^/K^+^ (**C**) in shoots of *C. maritima* plants grown from non-primed (NPr) and primed seeds (WPr, 200Pr, 400Pr) under different NaCl treatments (CK, 200 mM, and 400 mM NaCl) for 7 days. Values are presented as the mean ± SE (n = 6). Different letters indicate significant differences (Tukey test, *p* < 0.05).

Some of the key growth-limiting factors for plants in saline environments, apart from water stress, include Na^+^ and Cl⁻ toxicity and the associated deficiency of K^+^ and Ca^2+^ [34]. The most notable difference in shoot Na⁺ accumulation was observed between NPr and Pr plants, with primed plants exhibiting slightly lower Na⁺ values (Figure 3A). In the NPr plants, shoot Na⁺ levels increased significantly under both salt treatments compared to the control conditions, suggesting that priming mitigates Na⁺ accumulation as salinity increases.

In contrast, K^+^ levels were higher in the NaCl-primed plants than in the NPr and WPr plants in both CK and salt conditions (Figure 3B). However, 400 mM NaCl-primed plants showed a significant reduction in shoot K⁺ levels under both salt treatments compared to control conditions. These results indicate that the shoot K⁺ concentration is influenced by both priming and salinity, with potential trade-offs between Na⁺ exclusion and K⁺ retention mechanisms.

While the Na⁺/K⁺ ratio (Figure 3C) was not significantly affected by salinity, it was influenced by priming, with clear differences between Pr and NPr plants. The highest Na⁺/K⁺ ratio among priming groups was consistently observed under control conditions. This reinforces the role of halopriming in reducing Na⁺ accumulation and preventing K^+^ deficiency, thereby enhancing salt tolerance in *C. maritima* plants.

**Figure 4:**
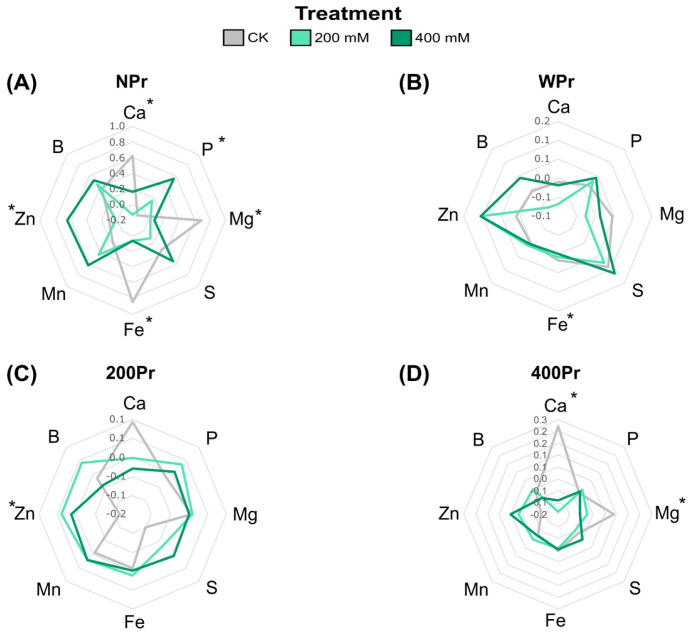
Radial plots showing shoot normalized mineral nutrient differences of *C. maritima* plants grown from non-primed (NPr) (**A**) and primed seeds (WPr (**B**), 200Pr (**C**), 400Pr (**D**)) under different NaCl treatments (CK, 200 mM, and 400 mM NaCl) for 7 days. Axes display Z-scores calculated per element (n = 6). Asterisks indicate significant differences in at least one treatment (Tukey test, *p* < 0.05).

Macronutrients such as Ca^2^⁺, Mg^2^⁺, P, and S, as well as micronutrients including Fe, Zn, Mn, and B, were also analysed in both salt-treated and salt-primed plants in roots and shoots. In shoots from NPr seeds, Ca^2^⁺, Mg^2^⁺, and Fe levels significantly decreased with increasing salinity, while P and Zn levels increased (Figure 4A). This Fe reduction agrees with the low soil availability of Fe under high levels of Na^+^ [35]. The NPr and WPr plants showed significant differences in Fe levels in the CK conditions compared to the salt treatments, while the haloprimed plants did not. In contrast, plants from the 400Pr seeds showed a decrease in Ca^2^⁺ levels under a higher salinity, particularly at the highest NaCl concentration, compared to control conditions (Figure 4D). Zinc, an essential micronutrient in all biological systems, increased in all plants under salinity conditions except for NPr seeds at 200 mM NaCl. Some researchers suggest that Zn plays a critical role in the metabolism of reactive oxygen species and in the function of Zn-finger proteins, which regulate defence responses and signalling [36]. This suggests that higher Zn levels in *C. maritima* plants could play a key role in mitigating oxidative damage induced by salinity, potentially enhancing the plant’s ability to cope with salt-induced stress. The magnesium levels showed contrasting results, in which a significant reduction was observed under the NaCl treatments in both the NPr and 400Pr conditions. Sulphur, B, and Mn content was not significantly affected by priming or salt treatment. Seed priming appeared to mitigate nutrient reductions, although no significant differences were detected between Pr and NPr seeds. Non-primed seeds showed a fluctuation in P levels when exposed to salinity, while primed groups were able to maintain a stable phosphate content at increasing salt concentrations (Figure 4C,D).

In roots, the NPr plants exhibited the highest nutrient content, showing the most significant differences between treatments for most elements. In contrast, the Pr plants had a lower overall nutrient content, which reduced the magnitude of differences among treatments (Figure S1).

### 3.3. Oxidative Status

**Figure 5:**
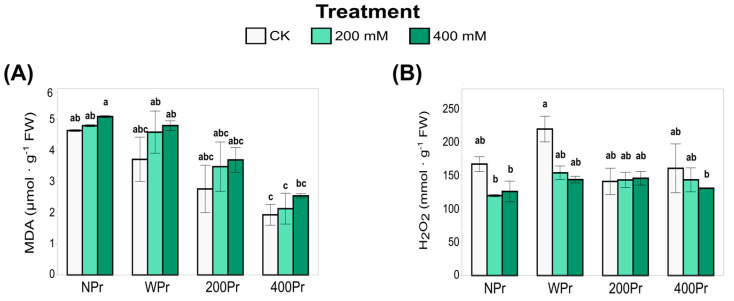
Concentration of malondialdehyde (**A**) and hydrogen peroxide (**B**) in shoots of *C. maritima* plants grown from non-primed (NPr) and primed seeds (WPr, 200Pr, 400Pr) under different NaCl treatments (CK, 200 mM, and 400 mM NaCl) for 7 days. Values are presented as the mean ± SE (n = 6). Different letters indicate significant differences (Tukey test, *p* < 0.05).

The oxidative status of *C. maritima* under salt stress was evaluated by analysing lipid peroxidation and hydrogen peroxide accumulation. Malondialdehyde (MDA), a marker of lipid peroxidation and oxidative damage, increased with increasing salinity in all priming groups, although these differences were not statistically significant (Figure 5A). Despite this overall increase, MDA levels tended to be lower in Pr plants compared to NPr ones, particularly in haloprimed plants. Among Pr plants, WPr exhibited the highest MDA values, followed by 200Pr and 400Pr. Notably, MDA levels in 400Pr were significantly lower than those in NPr and WPr under the control and low-NaCl conditions. These findings suggest that NaCl priming helps mitigate oxidative damage caused by salt stress, possibly by enhancing antioxidant defences or improving ion homeostasis.

Hydrogen peroxide (H_2_O_2_) plays a dual role as both an oxidative stress marker and a signaling molecule. H_2_O_2_ accumulation in the shoots of *C. maritima* did not vary significantly with priming or salt treatment (Figure 5B). However, a more pronounced, though not statistically significant, reduction in H_2_O_2_ content was observed in the NPr and WPr plants exposed to salt treatment compared to control conditions. Under control conditions, the NaCl-primed plants tended to have lower H_2_O_2_ levels than the NPr and WPr plants, suggesting a better-regulated ROS homeostasis. This balance may help prevent excessive oxidative damage while maintaining H_2_O_2_’s signaling function, which is essential for stress response and adaptation under saline conditions. The relationship between oxidative markers (MDA and H_2_O_2_) and antioxidant responses highlights the potential role of NaCl priming in promoting ROS homeostasis and enhancing stress tolerance.

### 3.4. Total Antioxidant Capacity and Antioxidant Enzyme Activity

**Figure 6:**
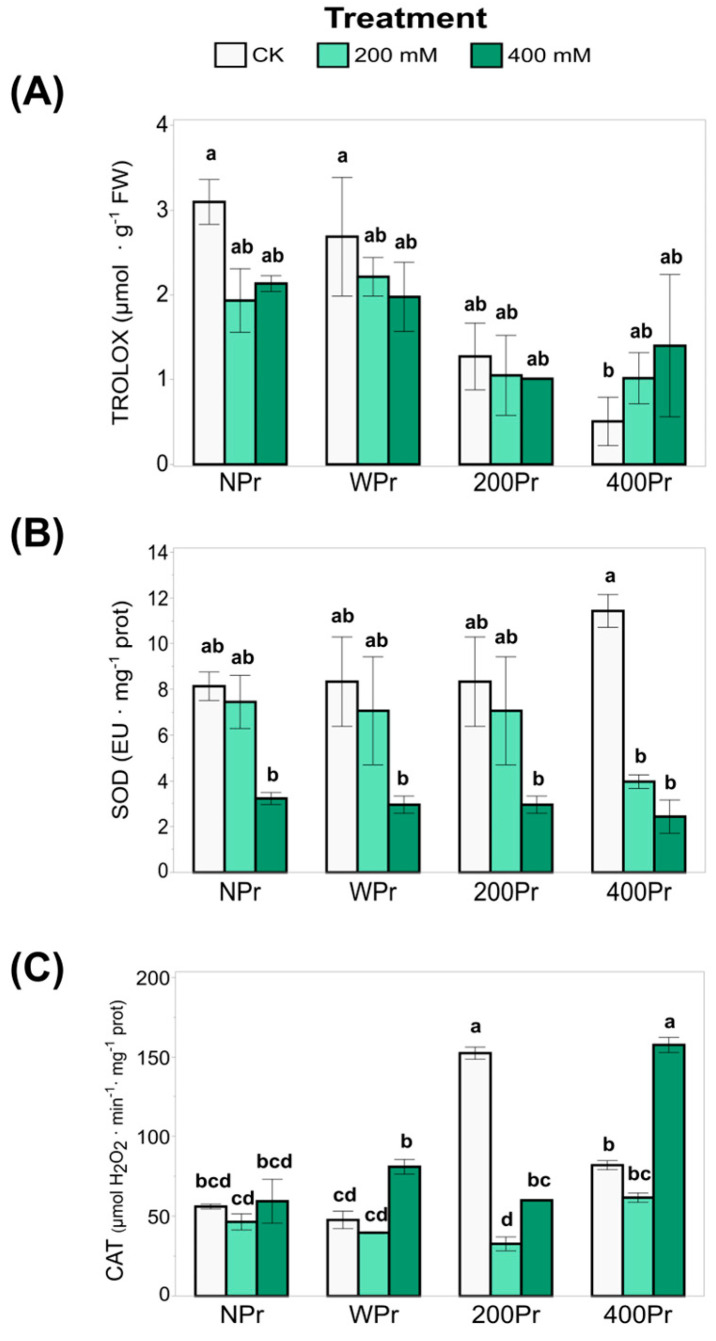
The total antioxidant capacity (**A**), SOD activity (**B**), and CAT activity (**C**) in shoots of *C. maritima* plants grown from non-primed (NPr) and primed seeds (WPr, 200Pr, 400Pr) under different NaCl treatments (CK, 200 mM, and 400 mM NaCl) for 7 days. Values are presented as the mean ± SE (n = 6). Different letters indicate significant differences (Tukey test, *p* < 0.05).

The total antioxidant capacity (TEAC), which reflects the ability to counteract oxidative stress in very small quantities (<1%, commonly 1–1000 mg L^−1^), was also analysed (Figure 6A). The highest TEAC values were observed in the WPr plants, showing an increase with rising salinity. A similar trend was noted in the 400Pr plants, in which a significant increase was observed under the 400 mM NaCl treatment compared to the other conditions, which showed no significant differences. Conversely, the lowest TEAC value was recorded in plants from seeds primed with 200 mM NaCl when exposed to 400 mM salinity. These results suggest that priming can modulate antioxidant capacity, but its effects depend on both the priming concentration and the level of salt stress. The decline in TEAC at 200 mM NaCl priming under severe salinity suggests that intermediate priming levels may not always provide enhanced oxidative protection, possibly due to an inadequate antioxidant response or a differential activation of defence mechanisms.

SOD activity, a key component of enzymatic antioxidant defence, decreased significantly with increasing salinity in all treatments, with the most pronounced reduction occurring under 400 mM NaCl (Figure 6B). While no significant differences were observed between seed priming groups or compared to NPr, SOD activity was notably reduced under a high salinity in the 400Pr plants. The highest SOD activity was recorded in control plants of the 400Pr group. This suggests that while SOD plays a key role in mitigating oxidative stress, its activity may be downregulated or overwhelmed under severe salinity. The decline in SOD activity at higher salt concentrations may indicate a shift towards other antioxidant mechanisms, such as non-enzymatic antioxidants, to compensate for this reduction in stressed plants.

Regarding CAT activity, plants from seeds primed with 200 mM NaCl and grown under control conditions exhibited increased CAT activity, suggesting a stimulatory effect (Figure 6C). However, at higher priming concentrations, CAT activity returned to levels comparable to the control. Under salinity stress, a similar trend was observed in the WPr and 400Pr groups, with a decrease in CAT at 200 mM NaCl followed by an increase at 400 mM NaCl. Interestingly, a distinct response was observed in the 200Pr group, in which the CAT activity dropped sharply in the 200 mM salinity treatment, resulting in the lowest recorded values. This pattern supports the hypothesis of an “imprinted memory” effect, in which initial exposure to specific conditions influences the plant’s later responses. The fluctuating CAT activity across treatments suggests a complex regulatory mechanism in which priming-induced changes in antioxidant enzyme activity do not always follow a linear pattern but instead depend on the severity of stress and the plant’s adaptive responses. We suggest from our results that NaCl priming modulates oxidative balance through multiple pathways, as there could be an interplay between enzymatic and non-enzymatic antioxidant responses that may explain the enhanced stress tolerance observed under specific priming conditions.

### 3.5. Effects of Seed Priming on Gene Expression

**Figure 7:**
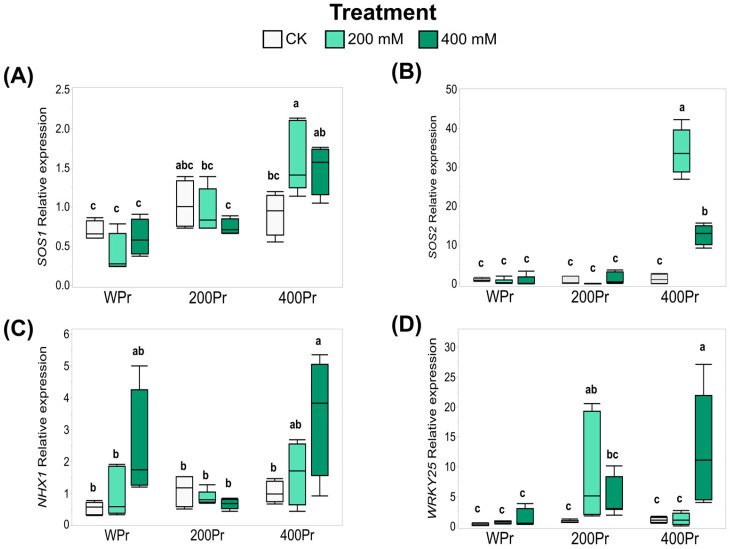
Relative expression levels of *CmSOS1* (**A**), *CmSOS2* (**B**), *CmNHX1* (**C**), and *CmWRKY25* (**D**) in shoots of *C. maritima* plants grown from non-primed (NPr) and primed seeds (WPr, 200Pr, 400Pr) under different NaCl treatments (CK, 200 mM, and 400 mM NaCl) for 7 days. Values are presented as the mean ± SE (n = 6). Different letters indicate significant differences (Tukey test, *p* < 0.05).

Based on the effects of seed priming on Na^+^ accumulation and the modification of oxidative stress in plants, the expression of key genes—*CmSOS1*, *CmSOS2*, *CmNHX1*, and *CmWRKY25*—was evaluated using RT-qPCR. The seeds primed with 400 mM NaCl showed an increased expression of the Na^+^/H^+^ transmembrane transporter *CmSOS1* when exposed to 200 mM NaCl. However, under 400 mM NaCl, the induction was more moderate and did not show significant differences compared to the control (Figure 7A). Similarly, the expression of *CmSOS2*, an SOS1-associated kinase, followed a pattern akin to *CmSOS1*, with a drastic increase observed at 400 mM NaCl priming under both the 200 mM and 400 mM NaCl treatments (Figure 7A,B).

For the vacuole Na^+^/H^+^ antiporter CmNHX1, the gene expression was induced under the 400 mM NaCl treatment, both in the WPr and 400Pr plants. However, this induction was not observed in plants from seeds primed with 200 mM NaCl (Figure 7C).

The expression of the stress-related transcription factor CmWRKY25 showed a different response across the various salt treatments, depending on the seed priming status. Water priming did not alter *CmWRKY25* expression under any treatment. In contrast, 200Pr plants exhibited a higher expression of *CmWRKY25* when grown under the same NaCl concentration. A similar pattern was observed for the 400Pr plants, in which an increase in expression was noted only under the 400 mM NaCl treatment (Figure 7D).

These findings suggest that seed priming, particularly with higher NaCl concentrations, primes plants for better adaptation to salt stress by modulating the expression of critical transporters and stress-related genes.

## 4. Discussion

Soil salinization is a major challenge that significantly impacts global agricultural productivity. Plants rely on their ability to perceive and respond to environmental stress for survival, but increasing soil salinity complicates crop establishment, reduces yields, and poses severe threats to food security and economic stability. In this context, seed priming has emerged as a promising technique to enhance plant resilience, performance, and productivity in saline soils. This study examines the effects of seed priming on the physiological, biochemical, and molecular responses of *C. maritima* under salt stress, testing the hypothesis that priming confers a ‘stress memory’ that enhances adaptation and productivity under increasing salinity. The findings reveal that NaCl priming influences germination, growth, water status, ion accumulation, oxidative stress responses, and gene expression in a concentration-dependent manner.

According to J. Levitt [14], plants must first be exposed to mild salt stress to develop tolerance to more severe salinity conditions. NaCl, when used as a priming agent, has been shown to enhance salt tolerance in various species. Seed priming can improve performance under both normal and saline conditions, promoting better germination, growth, and survival. The ability to recover from seed germination is a key adaptive trait for the successful establishment and dispersal of plants in extreme environments. *C. maritima*, a common annual halophyte along the Mediterranean coast, produces seeds with transient dormancy under high salinity and germinates rapidly when soil salinity decreases due to rainfall. In our results, seed priming significantly influenced germination rates and plant biomass under salt stress. While the control conditions showed nearly 100% germination in all treatments, salt stress drastically reduced germination rates, particularly in primed seeds at higher NaCl concentrations. This suggests that although priming may enhance germination under non-stressful conditions, its efficacy diminishes at extreme salinity levels. These fundings support a previously proposed hypothesis [37], which suggests that avoiding salinity during germination and establishment under high-salinity conditions serve as a survival strategy in *C. maritima*. However, our study also revealed that extreme salinity conditions can mimic priming effects in this species, aiding in the maintenance of seed germination and vigour under stress, albeit at lower levels than in control conditions, particularly at the highest salt concentrations. This effect is likely due to the activation of metabolic processes during priming, enhancing the plant’s ability to cope with salinity stress.

Shoot biomass data further support the role of priming in improving early growth, particularly under moderate stress levels. Haloprimed plants exhibited higher biomass under control conditions, while under a high salinity, non-primed seeds exhibited the lowest biomass. This highlights a potential trade-off between stress tolerance and early vigour, which may depend on the priming concentration and severity of stress. Several studies have demonstrated that primed plants exhibit significant improvements in growth, yield, and cost–benefit ratios, particularly under drought and salt stress conditions [6,38].

Salinity restricts water absorption in plants, leading to osmotic stress and ionic toxicity due to excessive metabolite accumulation. This decline in cellular water status influences turgor pressure, disrupts membrane stability, and contributes to biomass loss [39].

In *C. maritima* plants, water relations were also positively influenced by priming, as indicated by the higher relative water content in haloprimed plants under control conditions. Under salt stress, however, RWC declined in all treatments, with no significant differences among priming groups. This suggests that although priming improves initial water retention, its benefits may be masked under prolonged exposure to high salinity.

Interestingly, the photosynthetic efficiency, assessed through the Fv/Fm ratio (a widely used indicator of stress-induced alterations in the photosynthetic system [40]), remained largely unaffected by either salt stress or priming, with most values close to 0.8. This suggests that *C. maritima* maintains photosynthetic integrity under salinity, likely due to its inherent stress tolerance mechanisms.

The stomata play a crucial role in regulating water vapor and CO_2_ exchange, and their density is highly sensitive to environmental stressors [41]. *C. maritima* plants grown from 400Pr seeds exhibited a significant increase in stomatal density in the basal leaves, compared to untreated seeds (Table 2). This suggests that NaCl priming may help maintain better stomatal regulation, potentially enhancing the plant’s ability to cope with salt stress. Interestingly, mid-height leaves exhibited a more pronounced reduction in SD as the NaCl concentration increased, particularly in WPr plants. However, plants primed with 400 mM NaCl (400Pr) maintained a relatively high SD, suggesting that NaCl priming could be beneficial for stomatal maintenance in regions of the plant exposed to higher-salinity conditions. This result contrasts with findings in many other species, such as *Chenopodium quinoa* [41] and *Amaranthus* spp. [42], in which a decrease in stomatal density under salt stress is often considered a protective response to minimize water and salt uptake, thereby reducing the risk of toxic ion accumulation and dehydration [43]. NaCl priming in *C. maritima* appears to serve as an adaptive mechanism that not only maintains a high stomatal density but also likely supports better stomatal function in basal leaves. This could enable the plant to optimize photosynthetic efficiency and water regulation under high-salinity conditions, improving its overall salt tolerance.

Salinity stress significantly influences the nutrient balance and physiological responses of *C. maritima*, affecting both macro- and micronutrient concentrations. Our findings indicate that salinity reduces the availability of essential nutrients, leading to decreased plant dry mass and relative water content. However, primed plants exhibited a higher final water content, suggesting possible metabolic activation induced by the priming treatment, which may enhance salt tolerance.

Macronutrients such as Ca^2^⁺, Mg^2^⁺, P, and S exhibited differential accumulation patterns in response to salinity and seed priming. In non-primed (NPr) plants, Ca^2^⁺, Mg^2^⁺, and Fe levels significantly decreased with increasing salinity, whereas P and Zn levels increased. The observed decrease in Ca^2^⁺ and Mg^2^⁺ may be linked to competition with Na⁺ for uptake sites and the potential displacement of these cations in plant tissues. However, P levels increased in non-primed plants, which could be an adaptive response to maintain energy metabolism under stress conditions. Interestingly, primed (Pr) plants showed a decrease in Ca^2^⁺ levels at a higher salinity, particularly at the highest NaCl concentration. This suggests that NaCl priming does not fully mitigate Ca^2^⁺ displacement but may still aid in maintaining overall nutrient homeostasis. Additionally, while S, B, and Mn concentrations remained relatively stable across treatments, the increase in P levels in WPr plants suggests a potential priming-induced enhancement of P uptake.

Na⁺ and K⁺ homeostasis is a crucial determinant of plant salt tolerance. Our results confirm that *C. maritima* acts as a Na⁺-accumulating halophyte, efficiently compartmentalizing Na⁺ in leaves while maintaining lower concentrations in roots. The Na⁺/K⁺ ratio in leaves increased in primed plants with the salt treatment, indicating that the primed *C. maritima* employs an efficient translocation mechanism to transport Na⁺ from roots to shoots, avoiding generating oxidative stress. Notably, the Na⁺ levels in roots were nearly three times lower than in leaves at 400 mM NaCl, suggesting an effective sequestration strategy that prevents excessive ion accumulation in root tissues (Figure S2).

Iron was one of the most affected micronutrients, displaying significant reductions under salinity stress, particularly in non-primed plants, and importantly, NaCl priming did not enhance its absorption. The low Fe levels may be attributed to its reduced availability at a high pH rather than the direct impact of salinity. In natural soils in which *C. maritima* thrives, only about 1.2% of the total Fe is available for plant uptake.

Zinc, a vital micronutrient and the second most abundant metal cofactor after Fe [44], increased in all plants under saline conditions. Zn is known to influence various plant-specific functions, such as auxin regulation, photosystem II restoration, and CO_2_ stabilization in mesophyll tissues [45]. Zn also plays a pivotal role in reducing Na⁺ uptake and improving the K⁺/Na⁺ ratio, possibly by stabilizing membrane integrity and permeability [46]. This mechanism contributes to enhanced salt tolerance by preventing excessive Na⁺ accumulation and supporting ionic homeostasis. Additionally, Zn plays a crucial role in reactive oxygen species (ROS) metabolism and Zn-finger proteins, which regulate defence responses and signaling [36]. Elevated Zn levels in *C. maritima* could enhance antioxidant enzyme activity, such as SOD, CAT, and peroxidase (POD) activity, which strengthens the plant’s antioxidant defence system [47], thereby mitigating oxidative damage induced by salinity stress. These protective effects reinforce the hypothesis that Zn accumulation is a key adaptive strategy in *C. maritima* under saline conditions.

One of the most prominent responses to salinity stress in plants is the accumulation of reactive oxygen species (ROS). When produced in controlled amounts within specific cellular compartments, ROS play crucial roles in plant growth and development. However, excessive ROS production leads to uncontrolled oxidation, resulting in cellular damage and, ultimately, cell death. To mitigate this, plants activate antioxidant defences to regulate ROS levels and prevent damage, allowing their beneficial functions to continue [48]. In this study, malondialdehyde (MDA) and hydrogen peroxide (H_2_O_2_) levels were analysed as indicators of lipid peroxidation and oxidative stress, respectively. MDA, a product of lipid peroxidation, accumulates under stress conditions and is used as a marker of oxidative damage [49]. A high MDA level correlates with oxidative damage and free radical production in cell membranes, indicating the plant is susceptible to salinity stress. Conversely, a low MDA concentration suggests effective protection against oxidative stress, indicating the plant’s antioxidant defences are active and functioning under stressful conditions [50]. Many plants, especially under drought and salt stress, show improved growth and yield by maintaining high levels of soluble compounds and antioxidant enzymes, which help prevent lipid peroxidation and keep MDA and ROS levels in check [38,51]. These effects have been associated with lower oxidative damage and a better water balance in plants like chickpea and broad bean (*Vicia faba*). In our study, MDA levels increased in all priming groups as the NaCl concentration increased, with the highest values observed in the WPr treatment compared to the 200Pr and 400Pr treatments. Interestingly, the MDA concentration decreased with increasing NaCl priming concentration across the treatment groups.

Hydrogen peroxide (H_2_O_2_) acts as a secondary messenger in plants, playing a key role in regulating physiological responses to stress. Under salinity, controlled H_2_O_2_ levels can activate defence mechanisms, such as antioxidant enzyme activity, osmoprotectant accumulation, and stress-responsive gene expression. Our findings suggest that NaCl priming modulates H_2_O_2_ levels, reducing excessive oxidative damage while maintaining its role in stress signaling. This balance may contribute to enhanced salinity tolerance in Pr plants, which can better regulate their oxidative state while still triggering adaptive responses. Notably, the observed reduction in H_2_O_2_ levels in Pr plants suggests that priming enhances the plant’s ability to regulate ROS signaling more efficiently. Instead of allowing uncontrolled oxidative damage, primed plants likely use H_2_O_2_ in a controlled manner to fine-tune stress responses, thus promoting better acclimation to salinity. This aligns with the notion that moderate ROS production is not necessarily harmful but can help plants “prepare” for tougher conditions by activating protective mechanisms like antioxidant enzyme production and osmoprotectant accumulation. According to reports on other Brassicaceae halophytes such as *Eutrema halophilum* [52] and *Lepidium latifolium* L. [53], our results show that H_2_O_2_ levels decreased in the leaves of the WPr and the 400 mM NaCl plants, while they remained unchanged in the 200 mM NaCl medium-salinity treatment. This suggests that the salt priming treatment had a slightly positive effect, reducing peroxide accumulation compared to the WPr treatment, indicating that seed priming enhances antioxidant activity.

The extent of damage due to oxidative stress can also be addressed at the enzymatic level. In our study, we observed that H_2_O_2_ accumulation under the salt treatment was correlated with CAT activity in *C. maritima* across priming treatments. The CAT activity was upregulated, especially in the 400 mM NaCl treatment, in which peroxide levels were low. The activation of this enzyme is one of the key mechanisms for salt adaptation in halophytes under a high salinity [54]. In fact, plants possess a complex antioxidant enzyme system, with SOD and CAT playing critical roles in mitigating oxidative stress caused by ROS. Our findings show that CAT activity affected the rate of H_2_O_2_ radicals in the 400 mM NaCl treatment, helping to scavenge harmful free radicals. In contrast, the 200 mM NaCl treatment suggests that NaCl priming enhances salt tolerance at concentrations greater than 200 mM NaCl. The stimulatory effect observed at 200 mM NaCl appears to indicate that CAT activity is enhanced by salt stress and that seed priming imparts a “memory” trait to the seedling. Similar enhancements in antioxidant enzyme activity with increased NaCl concentrations have been reported in other halophyte species [20]. These findings indicate that NaCl priming enhances the plant’s ability to regulate oxidative stress by reducing both H_2_O_2_ and MDA accumulation, likely by activating antioxidant defence mechanisms and improving stress tolerance to high salinity in the halophyte *C. maritima*.

SOD is the only enzyme in plants that catalyses the dismutation of superoxide into oxygen and hydrogen peroxide, which can then be directly catabolized by CAT to produce oxygen and water. These two enzymes are among the most important antioxidant defences in cells exposed to oxygen. Maksimović et al. (2013) [55] reported that halophytes possess exceptional abilities to use SOD to protect themselves from extreme environmental changes. In *C. maritima*, the activity of both SOD and CAT enzymes is closely related to the plant’s response to saline stress. An increase in antioxidant enzyme activity indicates that the plant can reduce oxidative damage under these stress conditions. Ellouzi et al. (2011) [20] specifically found that effective SOD and CAT activities in *C. maritima* leaves allowed for a reduction in H_2_O_2_ levels over a prolonged period (72 h after salt treatment). Houmani et al. (2016) [23] studied SOD isozymes under long-term high-salinity conditions (400 mM NaCl) in *C*. *maritima*, showing that SOD activity increased under salt treatment, particularly CuZn-SODs, which were only detected during plant development and under severe salinity stress conditions. Moreover, in a study with 30-day-old *C*. *maritima* plants under K^+^ deficiency, oxidative stress was induced, leading to an overall increase in antioxidant activity, particularly in CuZn-SOD [56]. Our results show that haloprimed plants accumulate more K^+^ than NPr and WPr plants under both control and salinity conditions, with K^+^ levels consistently above deficiency thresholds. The optimal K^+^ levels, combined with the halopriming treatment of the seeds in our *Cakile* plants, could explain why the SOD activity did not increase under any salt treatment in these plants, even at the highest concentration (400 mM NaCl). Through its activity, SOD protects cells from damage caused by free radicals by modulating the levels of O_2_^−^ and H_2_O_2_, maintaining physiological balance by managing oxidative stress and intracellular ROS levels. As H_2_O_2_ is the end-product of SOD activity, comparing the H_2_O_2_ accumulation pattern in halophytes and glycophytes may provide valuable insights into ROS signaling and damage. This comparison clearly suggests that halophytes are quick to send stress signals via H_2_O_2_ and possess efficient antioxidant mechanisms to scavenge H_2_O_2_ once signaling is completed [54]. However, SOD is closely intertwined with other enzymes and antioxidants, such as ascorbate, glutathione, alpha-tocopherol, and carotenoids, in what is likely a finely tuned balance that reduces the risk of hydroxyl radical formation. Therefore, when discussing the role of SOD, it is crucial to consider the entire oxidative stress defence system [57]. Additionally, the plant’s total response to salt stress may result from the activation of several metabolic pathways working in tandem.

The antioxidant capacity of plants has been recognized for centuries, as they contain compounds that act as reducing agents, inhibiting the oxidation of other molecules. Plants under salt stress are known to accumulate a wide variety of secondary metabolites, which act as antioxidants to protect the plant from oxidative damage caused by light, temperature, drought, salinity, and other stressors [58]. Depending on the severity of salt stress, these metabolites can accumulate or decrease in the plant to help protect the cell from the dehydration and osmotic stress caused by high salt concentrations. In fact, an increase in antioxidant production helps the plant withstand environmental stress requiring a comprehensive approach to analysis. As a first step in this study, we assessed the TEAC to determine whether seed priming could enhance the antioxidant response in *C. maritima* under different salt concentrations. The highest TEAC values were observed in plants grown from WPr seeds, with a progressive increase under high-salinity conditions (Figure 6A). A similar trend was observed in the 400Pr plants, with significant differences detected at 400 mM NaCl compared to the other treatments, which showed no significant differences. In general, priming treatments appear to reduce TEAC levels in all cases compared to WPr. Interestingly, plants primed with 200 mM NaCl exhibited a distinct response under the highest salinity condition, suggesting that the memory of the initial exposure to 200 mM NaCl may enhance the plant’s ability to cope with subsequent high salinity levels, thereby mitigating the effects of severe salt stress.

Some halophytes, including *C. maritima*, can accumulate large concentrations of Na^+^ in their leaves when exposed to salinity, indicating efficient Na^+^ translocation from roots to shoots. This rapid translocation is crucial for maintaining a toxic-free root zone, ensuring proper root function under saline conditions. To regulate ionic homeostasis, plants rely on the Salt Overly Sensitive (SOS) signaling pathway, which comprises three key components responsible for interpreting salt-induced Ca^2+^ signals and safeguarding the plant against salt stress. Among them, SOS*1* encodes a plasma membrane Na^+^/H^+^ antiporter that plays a key role in Na^+^ extrusion and the regulation of long-distance Na^+^ transport from roots to shoots. SOS3 encodes an EF-hand Ca^2+^-binding protein that functions as a Ca^2+^ sensor for salt tolerance and SOS2 encodes a Ser/Thr protein kinase [59]. These key membrane transporters, such as the salt-sensitive SOS1 and the Na^+^-hydrogen exchanger NHX1, have been found to help regulate Na^+^ efflux, and, together with the *SOS2* and *SOS3* genes, are required for K^+^ and Na^+^ ion homeostasis and salt tolerance in plants [60,61]. Under salt stress conditions, excess Na^+^ ions trigger a cytosolic Ca^2+^ signal, which is mediated by *SOS3*. This last gene interacts with *SOS2* and the activated SOS2/SOS3 kinase complex phosphorylates and activates *SOS1* and *NHX* to reduce the cytosolic Na^+^ concentration [62,63]. Although *SOS3* is primarily expressed in roots, the binding protein (SCaBP8) with which it interacts under salt stress is expressed mainly in the shoots. When *SOS3* and SCaBP8 bind, they activate *SOS2*, which in turn activates *SOS1* at the plasma membrane, facilitating Na^+^ exclusion from the cytoplasm into the apoplast [64]. In our study, an increased expression of *SOS1* was observed in response to 200 mM NaCl, with a milder increase under 400 mM NaCl in the 400Pr group. This highlights the lasting effect of seed priming, which modifies gene expression even days after exposure to stress. A similar pattern was detected for *CmSOS2*, in which only plants derived from seeds primed with 400mM NaCl showed an increased expression of these genes under salt stress. Notably, *SOS1* overexpression following seed priming with water or PEG has been previously reported in *Hibiscus cannabinus* seedlings subjected to 150 mM NaCl for 7 days [65].

The expression of the vacuolar Na^+^/H^+^ transporter *NHX1*, which facilitates the sequestration of Na^+^ into the vacuole to maintain cellular ion homeostasis, was also influenced by seed priming. Notably, its expression increased under the 400 mM NaCl treatment in both the WPr and 400Pr groups, whereas no significant change was observed in the 200Pr group. Similarly, increased *NHX1* expression has been reported in salt-stressed sugarcane plants after priming the seeds with NaCl, further supporting our findings [66].

Additionally, the WRKY transcription factor family plays a key role in modulating stress responses, such as tolerance to oxidative stress, the regulation of intracellular redox conditions, and the control of senescence [67]. In *Arabidopsis*, microarray analyses identified two WRKY transcription factors, *WRKY25* and *WRKY33*, which increased in abundance after NaCl treatment. Specifically, the overexpression of *WRKY25* was sufficient to enhance NaCl tolerance in *Arabidopsis* [68]. In our study, the expression of *CmWRKY25* displayed a different expression pattern depending on both the salt treatment and seed priming condition. Notably, its induction appeared to occur only when the plants were exposed to a NaCl concentration equal to or greater than the one they were primed with, suggesting a potential memory effect of the initial priming treatment.

Future research is essential to determine whether laboratory findings can be replicated in real-world environmental conditions, as the benefits of seed priming tend to diminish after 15 days of storage [69]. Overcoming the poor storability of primed seeds remains a major challenge for long-term storage. This issue is critical as crop production losses and poor seed vigour can lead to instability and increased pressure on governments, a problem that is expected to worsen due to climate change. The United Nations in their Agenda 2030 for Sustainable Development [70] places a strong emphasis on food security and seed research, highlighting a significant gap in the widespread use of priming techniques at the commercial level. While more scientific knowledge is needed to overcome these challenges, seed halopriming presents a promising, effective, and cost-efficient strategy that could be adopted by farmers in the short term. The molecular pathways and mechanisms underlying seed priming-induced stress tolerance, particularly in the context of multiple stressors such as salinity, drought, heat, and nutrient deficiencies, must be further explored. Zinc has been shown to increase the expression of stress-responsive genes like *GmZF351* and *OsZFP* and to reduce Na^+^ uptake by enhancing the integrity and permeability of cell membranes [71]. Investigating how these processes interact with seed priming could provide deeper insights into improving salt tolerance. Additionally, the concept of “stress memory” induced by seed priming remains largely unexplored. Future studies should focus on how priming influences epigenetic modifications, gene expression, and protein synthesis, and how these changes might enable plants to “remember” stress, allowing them to exhibit enhanced resilience. Understanding these molecular processes will be critical for assessing the long-term effectiveness and limitations of seed priming as a strategy for improving stress tolerance in plants exposed to various environmental challenges.

## 5. Conclusions

The results of this study highlight the potential of seed priming as an effective strategy to enhance the resilience of *C. maritima* to salt stress. Seed halopriming improved germination rates, biomass production, and water retention, particularly under moderate-salinity conditions. However, the benefits of priming were less pronounced under extreme salt concentrations, suggesting that while priming provides adaptive advantages, its efficacy may be limited at high salinity levels. Notably, NaCl priming influenced the plant’s physiological responses, such as stomatal regulation, ion accumulation, and antioxidant activity, which play crucial roles in mitigating oxidative damage and maintaining cellular homeostasis. The study also underscores the importance of the plant’s ability to modulate oxidative stress through enzyme activation, such as CAT and SOD, and through the accumulation of essential nutrients like Zn, which further enhances its salt tolerance. Additionally, molecular responses involving key genes of transporters and transcription factors, such as *CmSOS1*, *CmNHX1*, and *CmWRKY25*, were activated, supporting the hypothesis that priming can induce a ‘memory’ effect, enabling the plant to better cope with future salt stress. Overall, seed priming with NaCl appears to activate metabolic pathways that enhance the plant’s tolerance to salinity, though the degree of effectiveness varies depending on the concentration of salt and duration of exposure.

## Figures and Tables

**Table 1:** Primers used for RT-qPCR analysis. Gene accession number corresponds to the gene identifier described in Phytozome database.

Name	Gene Accession Number	Forward Primer (5′->3′)	Reverse Primer (5′->3′)
*NHX1*	*Camar.0770s0019*	GCT ACT GGT CTG ATA AGT GC	GCC AGG TGT AAT GGG ACA TC
*SOS1*	*Camar.0267s0003*	TCT GAA CGA GCA AGG CAA CT	GCT TTC TGA TTT CGC TGC GT
*SOS2*	*Camar.3181s0008*	ACG TTA GAA AGG CTG CTG GT	TGA CCT GCC TGA ATC AAA ACG
*WRKY25*	*Camar.2001s0011*	TCG CCT TCT CCG ATT TGC TT	CGG TTG CGT TTG TAG ATG CC
*UBQ10*	*Camar.1635s0021*	AAC CTC TTC TCC TTC ACA AC	CGT TGT CGA TGG TGT CAG AG

**Table 2:** Stomatal density percentage relative to water priming (WPr) and control (CK) conditions as the reference.

Priming	Treatment (NaCl)	Basal Leaves	Mid-Height Leaves
WPr	CK	100.0%	100.0%
WPr	200 mM	85.2%	63.0%
WPr	400 mM	110.5%	66.6%
200Pr	CK	118.7%	56.3%
200Pr	200 mM	115.3%	54.0%
200Pr	400 mM	75.2%	55.5%
400Pr	CK	83.3%	51.7%
400Pr	200 mM	84.7%	65.4%
400Pr	400 mM	131.0%	82.6%

## Data Availability

The original contributions presented in this study are included in the article. Further inquiries can be directed at the corresponding authors.

## Supplementary Materials

The following supporting information can be downloaded at: https://www.mdpi.com/article/10.3390/antiox14030353/s1, Dataset S1: Statistical results of the manuscript data analysis. Figure S1: Root mineral nutrient radial plot. Figure S2: Root Na+ concentration.

## Abbreviations

The following abbreviations are used in this manuscript:

CAT	Catalase
CC	Climate change
DPPH	2,2-Diphenyl-1-picrylhydrazyl
GP	Germination percentage
MDA	Malondialdehyde
NPr	Non-primed seeds/plants
Pr	Primed seeds/plants
ROS	Reactive oxygen species
RWC	Relative water content
SD	Stomatal density
SOD	Superoxide dismutase
TCA	Trichloroacetic acid
TEAC	Total antioxidant capacity
WPr	Water-primed seeds/plants
