# Supplementary material for: Seed Halopriming as an Effective Strategy to Enhance Salt Tolerance in Cakile maritima: Activation of Antioxidant and Genetic Responses
Source: Antioxidants (Basel). 2025 Mar 18;14(3):353. doi: 10.3390/antiox14030353 (PMC11939278; doi:10.3390/antiox14030353)
Supplement: Supplementary file 1 [file antioxidants-14-00353-s001.zip › antioxidants-3488099-supplementary.pdf]

% GERMINATION

Day 1, CK

Analysis of variance

Source	DF	Sum of Squares	Mean Square	F Ratio
Model	3	0	0	.
Error	8	0	0	**Prob > F**
C. Total	11	0		.

Seed priming Tukey HSD

Level	- Level	Difference	Std Err Dif	Lower CL	Upper CL	p-Value
WPr	NPr	0	0	0	0	.
200Pr	NPr	0	0	0	0	.
200Pr	WPr	0	0	0	0	.
400Pr	NPr	0	0	0	0	.
400Pr	WPr	0	0	0	0	.
400Pr	200Pr	0	0	0	0	.

Day 1, 200 mM NaCl

Analysis of variance

Source	DF	Sum of Squares	Mean Square	F Ratio
Model	3	0	0	.
Error	8	0	0	**Prob > F**
C. Total	11	0		.

Seed priming Tukey HSD

Level	- Level	Difference	Std Err Dif	Lower CL	Upper CL	p-Value
WPr	NPr	0	0	0	0	.
200Pr	NPr	0	0	0	0	.
200Pr	WPr	0	0	0	0	.
400Pr	NPr	0	0	0	0	.
400Pr	WPr	0	0	0	0	.
400Pr	200Pr	0	0	0	0	.

Day 1, 400 mM NaCl

Analysis of variance

Source	DF	Sum of Squares	Mean Square	F Ratio
Model	3	0	0	.
Error	8	0	0	**Prob > F**
C. Total	11	0		.

Seed priming Tukey HSD

Level	- Level	Difference	Std Err Dif	Lower CL	Upper CL	p-Value
WPr	NPr	0	0	0	0	.
200Pr	NPr	0	0	0	0	.
200Pr	WPr	0	0	0	0	.
400Pr	NPr	0	0	0	0	.

Level	- Level	Difference	Std Err Dif	Lower CL	Upper CL	p-Value
400Pr	WPr	0	0	0	0	.
400Pr	200Pr	0	0	0	0	.

Day 2, CK

Analysis of variance

Source	DF	Sum of Squares	Mean Square	F Ratio
Model	3	0.155	0.051	24.80
Error	8	0.016	0.002	**Prob > F**
C. Total	11	0.171		0.0002*

Seed priming Tukey HSD

Level	- Level	Difference	Std Err Dif	Lower CL	Upper CL	p-Value
NPr	200Pr	0.283	0.037	0.163	0.402	0.0003*
WPr	200Pr	0.233	0.037	0.113	0.352	0.0011*
NPr	400Pr	0.200	0.037	0.080	0.319	0.0030*
WPr	400Pr	0.150	0.037	0.030	0.269	0.0161*
400Pr	200Pr	0.083	0.037	-0.036	0.202	0.1932
NPr	WPr	0.050	0.037	-0.069	0.169	0.5647

Day 2, 200 mM NaCl

Analysis of variance

Source	DF	Sum of Squares	Mean Square	F Ratio
Model	3	0.090	0.030	20.57
Error	8	0.011	0.001	**Prob > F**
C. Total	11	0.101		0.0004*

Seed priming Tukey HSD

Level	- Level	Difference	Std Err Dif	Lower CL	Upper CL	p-Value
WPr	NPr	0.200	0.031	0.1001	0.299	0.0009*
WPr	200Pr	0.200	0.031	0.1001	0.299	0.0009*
400Pr	NPr	0.133	0.031	0.033	0.233	0.0115*
400Pr	200Pr	0.133	0.031	0.033	0.233	0.0115*
WPr	400Pr	0.067	0.031	-0.033	0.166	0.2203
NPr	200Pr	2.776e-17	0.031	-0.099	0.099	1.0000

Day 2, 400 mM NaCl

Analysis of variance

Source	DF	Sum of Squares	Mean Square	F Ratio
Model	3	0	0	.
Error	8	0	0	**Prob > F**
C. Total	11	0		.

Seed priming Tukey HSD

Level	- Level	Difference	Std Err Dif	Lower CL	Upper CL	p-Value
WPr	NPr	0	0	0	0	.
200Pr	NPr	0	0	0	0	.
200Pr	WPr	0	0	0	0	.
400Pr	NPr	0	0	0	0	.
400Pr	WPr	0	0	0	0	.
400Pr	200Pr	0	0	0	0	.

Day 3, CK

Analysis of variance

Source	DF	Sum of Squares	Mean Square	F Ratio
Model	3	0.1008	0.033	80.66
Error	8	0.003	0.0004	**Prob > F**
C. Total	11	0.104		<.0001*

Seed priming Tukey HSD

Level	- Level	Difference	Std Err Dif	Lower CL	Upper CL	p-Value
NPr	400Pr	0.217	0.0167	0.163	0.270	<.0001*
WPr	400Pr	0.217	0.0167	0.163	0.270	<.0001*
200Pr	400Pr	0.200	0.0167	0.146	0.2533724	<.0001*
NPr	200Pr	0.0167	0.0167	-0.036	0.070	0.7538
WPr	200Pr	0.0167	0.0167	-0.036	0.070	0.7538
WPr	NPr	0.000	0.0167	-0.053	0.053	1.0000

Day 3, 200 mM NaCl

Analysis of variance

Source	DF	Sum of Squares	Mean Square	F Ratio
Model	3	0.171	0.057	30.51
Error	8	0.015	0.001	**Prob > F**
C. Total	11	0.186		<.0001*

Seed priming Tukey HSD

Level	- Level	Difference	Std Err Dif	Lower CL	Upper CL	p-Value
400Pr	NPr	0.316	0.035	0.203	0.429	<.0001*
400Pr	200Pr	0.233	0.035	0.120	0.346	0.0008*
WPr	NPr	0.200	0.035	0.086	0.313	0.0021*
WPr	200Pr	0.116	0.035	0.003	0.229	0.0436*
400Pr	WPr	0.116	0.035	0.003	0.229	0.0436*
200Pr	NPr	0.083	0.035	-0.029	0.196	0.1638

Day 3, 400 mM NaCl

Analysis of variance

Source	DF	Sum of Squares	Mean Square	F Ratio
Model	3	0.143	0.046	115.16
Error	8	0.003	0.0004	**Prob > F**
C. Total	11	0.147		<.0001*

Seed priming Tukey HSD

Level	- Level	Difference	Std Err Dif	Lower CL	Upper CL	p-Value
400Pr	WPr	0.266	0.016	0.213	0.320	<.0001*
400Pr	NPr	0.266	0.016	0.213	0.320	<.0001*
400Pr	200Pr	0.150	0.016	0.096	0.203	<.0001*
200Pr	WPr	0.116	0.016	0.063	0.170	0.0005*
200Pr	NPr	0.116	0.016	0.063	0.170	0.0005*
NPr	WPr	2.776e-17	0.016	-0.053	0.053	1.0000

Day 4, CK

Analysis of variance

Source	DF	Sum of Squares	Mean Square	F Ratio
Model	3	0.002	0.0007	1.83
Error	8	0.003	0.0004	**Prob > F**
C. Total	11	0.005		0.2192

Seed priming Tukey HSD

Level	- Level	Difference	Std Err Dif	Lower CL	Upper CL	p-Value
NPr	400Pr	0.033	0.016	-0.020	0.086	0.2641
WPr	400Pr	0.033	0.016	-0.020	0.086	0.2641
NPr	200Pr	0.016	0.016	-0.036	0.070	0.7538
WPr	200Pr	0.016	0.016	-0.036	0.070	0.7538
200Pr	400Pr	0.016	0.016	-0.036	0.070	0.7538
WPr	NPr	0.000	0.016	-0.053	0.053	1.0000

Day 4, 200 mM NaCl

Analysis of variance

Source	DF	Sum of Squares	Mean Square	F Ratio
Model	3	0.285	0.095	76.16
Error	8	0.010	0.0012	**Prob > F**
C. Total	11	0.295		<.0001*

Seed priming Tukey HSD

Level	- Level	Difference	Std Err Dif	Lower CL	Upper CL	p-Value
400Pr	NPr	0.416	0.0288675	0.3242230	0.5091104	<.0001*
400Pr	200Pr	0.3166	0.0288675	0.2242230	0.4091104	<.0001*

Level	- Level	Difference	Std Err Dif	Lower CL	Upper CL	p-Value
400Pr	WPr	0.2166	0.028	0.124	0.309	0.0003*
WPr	NPr	0.200	0.028	0.107	0.292	0.0006*
200Pr	NPr	0.100	0.028	0.007	0.192	0.0347*
WPr	200Pr	0.100	0.028	0.007	0.192	0.0347*

Day 4, 400 mM NaCl

Analysis of variance

Source	DF	Sum of Squares	Mean Square	F Ratio
Model	3	0.146	0.048	117.33
Error	8	0.003	0.0004	**Prob > F**
C. Total	11	0.150		<.0001*

Seed priming Tukey HSD

Level	- Level	Difference	Std Err Dif	Lower CL	Upper CL	p-Value
400Pr	NPr	0.266	0.016	0.213	0.320	<.0001*
400Pr	WPr	0.266	0.016	0.213	0.320	<.0001*
400Pr	200Pr	0.133	0.016	0.079	0.186	0.0002*
200Pr	WPr	0.133	0.016	0.079	0.186	0.0002*
200Pr	NPr	0.133	0.016	0.079	0.186	0.0002*
NPr	WPr	1.388e-17	0.016	-0.053	0.053	1.0000

g DW

Analysis of variance

Source	DF	Sum of Squares	Mean Square	F Ratio
Model	11	0.59	0.05	13.03
Error	60	0.25	0.004	**Prob > F**
C. Total	71	0.85		<.0001*

Seed priming Tukey HSD

Level	- Level	Difference	Std Err Dif	Lower CL	Upper CL	p-Value
400Pr	NPr	0.13	0.02	0.07	0.19	<.0001*
200Pr	NPr	0.12	0.02	0.06	0.18	<.0001*
WPr	NPr	0.08	0.02	0.02	0.13	0.0034*
400Pr	WPr	0.054	0.02	-0.003	0.11	0.07
200Pr	WPr	0.043	0.02	-0.01	0.10	0.20
400Pr	200Pr	0.011	0.02	-0.04	0.07	0.96

Treatment Tukey HSD

Level	- Level	Difference	Std Err Dif	Lower CL	Upper CL	p-Value
CK	400 mM	0.15	0.019	0.11	0.20	<.0001*
CK	200 mM	0.11	0.019	0.07	0.16	<.0001*
200 mM	400 mM	0.04	0.019	-0.003	0.08	0.08

Seed priming * Treatment Tukey HSD

Level	- Level	Difference	Std Err Dif	Lower CL	Upper CL	p-Value
400Pr,CK	NPr,400 mM	0.37	0.04	0.24	0.49	<.0001*
200Pr,CK	NPr,400 mM	0.33	0.04	0.20	0.45	<.0001*
400Pr,CK	NPr,200 mM	0.27	0.04	0.14	0.39	<.0001*
400Pr,CK	200Pr,400 mM	0.23	0.04	0.11	0.36	<.0001*
200Pr,CK	NPr,200 mM	0.22	0.04	0.09	0.35	<.0001*
400Pr,CK	400Pr,400 mM	0.21	0.04	0.08	0.34	<.0001*
400Pr,CK	WPr,200 mM	0.21	0.04	0.08	0.33	<.0001*
400Pr,CK	400Pr,200 mM	0.20	0.04	0.07	0.33	<.0001*
200Pr,200 mM	NPr,400 mM	0.20	0.04	0.07	0.33	<.0001*
200Pr,CK	200Pr,400 mM	0.19	0.04	0.07	0.32	0.0001*
WPr,CK	NPr,400 mM	0.19	0.04	0.07	0.32	0.0001*
NPr,CK	NPr,400 mM	0.19	0.04	0.06	0.32	0.0002*
400Pr,CK	WPr,400 mM	0.19	0.04	0.06	0.30	0.0002*
WPr,400 mM	NPr,400 mM	0.17	0.04	0.05	0.30	0.0007*
400Pr,CK	NPr,CK	0.17	0.04	0.05	0.30	0.0009*
400Pr,CK	WPr,CK	0.17	0.04	0.04	0.30	0.0010*
200Pr,CK	400Pr,400 mM	0.17	0.04	0.04	0.29	0.0015*
200Pr,CK	WPr,200 mM	0.18	0.04	0.04	0.29	0.0017*
400Pr,CK	200Pr,200 mM	0.16	0.04	0.04	0.29	0.0020*
400Pr,200 mM	NPr,400 mM	0.16	0.04	0.04	0.29	0.0021*
200Pr,CK	400Pr,200 mM	0.16	0.04	0.03	0.28	0.0035*
WPr,200 mM	NPr,400 mM	0.16	0.04	0.03	0.28	0.0042*
400Pr,400 mM	NPr,400 mM	0.16	0.04	0.03	0.28	0.0046*
200Pr,CK	WPr,400 mM	0.15	0.04	0.02	0.27	0.0092*
200Pr,CK	NPr,CK	0.13	0.04	0.006	0.26	0.0323*
200Pr,CK	WPr,CK	0.13	0.04	0.005	0.26	0.0342*
200Pr,400 mM	NPr,400 mM	0.13	0.04	0.003	0.25	0.0387*
200Pr,CK	200Pr,200 mM	0.12	0.04	-0.002	0.25	0.0590
NPr,200 mM	NPr,400 mM	0.10	0.04	-0.02	0.26	0.2188
200Pr,200 mM	NPr,200 mM	0.01	0.04	-0.03	0.22	0.2820
WPr,CK	NPr,200 mM	0.09	0.04	-0.03	0.22	0.3991
NPr,CK	NPr,200 mM	0.09	0.04	-0.03	0.21	0.4115
WPr,400 mM	NPr,200 mM	0.07	0.04	-0.05	0.20	0.6974
200Pr,200 mM	200Pr,400 mM	0.07	0.04	-0.05	0.19	0.7452
WPr,CK	200Pr,400 mM	0.06	0.04	-0.06	0.19	0.8555
NPr,CK	200Pr,400 mM	0.06	0.04	-0.06	0.19	0.8645
400Pr,200 mM	NPr,200 mM	0.06	0.04	-0.06	0.19	0.8697
WPr,200 mM	NPr,200 mM	0.05	0.04	-0.07	0.18	0.9435
400Pr,400 mM	NPr,200 mM	0.05	0.04	-0.07	0.18	0.9519
WPr,400 mM	200Pr,400 mM	0.05	0.04	-0.08	0.17	0.9802
200Pr,200 mM	400Pr,400 mM	0.04	0.04	-0.08	0.17	0.9865
200Pr,200 mM	WPr,200 mM	0.04	0.04	-0.08	0.17	0.9893
400Pr,CK	200Pr,CK	0.04	0.04	-0.08	0.17	0.9920
WPr,CK	400Pr,400 mM	0.03	0.04	-0.09	0.16	0.9971
NPr,CK	400Pr,400 mM	0.03	0.04	-0.09	0.16	0.9975
WPr,CK	WPr,200 mM	0.03	0.04	-0.09	0.16	0.9979
200Pr,200 mM	400Pr,200 mM	0.03	0.04	-0.09	0.16	0.9980
400Pr,200 mM	200Pr,400 mM	0.03	0.04	-0.09	0.16	0.9981
NPr,CK	WPr,200 mM	0.03	0.04	-0.09	0.16	0.9982
WPr,CK	400Pr,200 mM	0.02	0.04	-0.09	0.15	0.9998
WPr,200 mM	200Pr,400 mM	0.03	0.04	-0.09	0.15	0.9998
NPr,CK	400Pr,200 mM	0.03	0.04	-0.10	0.15	0.9998
200Pr,400 mM	NPr,200 mM	0.02	0.04	-0.09	0.15	0.9999
400Pr,400 mM	200Pr,400 mM	0.02	0.04	-0.10	0.15	0.9999
200Pr,200 mM	WPr,400 mM	0.02	0.04	-0.10	0.15	1.0000
WPr,400 mM	400Pr,400 mM	0.02	0.04	-0.10	0.15	1.0000
WPr,400 mM	WPr,200 mM	0.02	0.04	-0.11	0.15	1.0000
WPr,CK	WPr,400 mM	0.01	0.04	-0.11	0.14	1.0000

Red values mean p value< 0.05; Orange values mean p value< 0.01

Level	- Level	Difference	Std Err Dif	Lower CL	Upper CL	p-Value
NPr,CK	WPr,400 mM	0.02	0.04	-0.11	0.14	1.0000
WPr,400 mM	400Pr,200 mM	0.01	0.04	-0.11	0.13	1.0000
400Pr,200 mM	400Pr,400 mM	0.01	0.04	-0.12	0.13	1.0000
200Pr,200 mM	NPr,CK	0.008	0.04	-0.12	0.13	1.0000
400Pr,200 mM	WPr,200 mM	0.008	0.04	-0.12	0.13	1.0000
200Pr,200 mM	WPr,CK	0.007	0.04	-0.12	0.13	1.0000
WPr,200 mM	400Pr,400 mM	0.001	0.04	-0.12	0.13	1.0000
WPr,CK	NPr,CK	0.0007	0.04	-0.12	0.13	1.0000

Relative Water Content (RWC)

Analysis of variance

Source	DF	Sum of Squares	Mean Square	F Ratio
Model	11	6187.82	562.53	22.90
Error	60	1473.25	24.55	**Prob > F**
C. Total	71	7661.07		<.0001*

Seed priming Tukey HSD

Level	- Level	Difference	Std Err Dif	Lower CL	Upper CL	p-Value
400Pr	NPr	20.37	1.65	16.01	24.74	<.0001*
200Pr	NPr	20.26	1.65	15.90	24.62	<.0001*
WPr	NPr	12.05	1.65	7.70	16.42	<.0001*
400Pr	WPr	8.32	1.65	3.95	12.69	<.0001*
200Pr	WPr	8.20	1.65	3.84	12.57	<.0001*
400Pr	200Pr	0.11	1.65	-4.25	4.48	0.9999

Treatment Tukey HSD

Level	- Level	Difference	Std Err Dif	Lower CL	Upper CL	p-Value
CK	400 mM	7.99	1.43	4.55	11.45	<.0001*
CK	200 mM	4.51	1.43	1.07	7.95	0.0069*
200 mM	400 mM	3.47	1.43	0.03	6.92	0.0470*

Seed priming * Treatment Tukey HSD

Level	- Level	Difference	Std Err Dif	Lower CL	Upper CL	p-Value
400Pr,CK	NPr,400 mM	32.46	2.86	22.73	42.19	<.0001*
200Pr,CK	NPr,400 mM	31.60	2.86	21.87	41.32	<.0001*
200Pr,200 mM	NPr,400 mM	29.28	2.86	19.55	39.01	<.0001*
400Pr,200 mM	NPr,400 mM	28.38	2.86	18.65	38.11	<.0001*
400Pr,400 mM	NPr,400 mM	25.11	2.86	15.38	34.84	<.0001*
200Pr,400 mM	NPr,400 mM	24.73	2.86	15.00	34.46	<.0001*
400Pr,CK	NPr,200 mM	24.70	2.86	14.97	34.42	<.0001*
200Pr,CK	NPr,200 mM	23.83	2.86	14.10	33.56	<.0001*
200Pr,200 mM	NPr,200 mM	21.51	2.86	11.78	31.24	<.0001*
WPr,CK	NPr,400 mM	21.35	2.86	11.62	31.07	<.0001*
WPr,400 mM	NPr,400 mM	20.66	2.86	10.93	30.39	<.0001*
400Pr,200 mM	NPr,200 mM	20.61	2.86	10.89	30.34	<.0001*
WPr,200 mM	NPr,400 mM	18.98	2.86	9.25	28.71	<.0001*
400Pr,400 mM	NPr,200 mM	17.35	2.86	7.62	27.07	<.0001*
NPr,CK	NPr,400 mM	17.06	2.86	7.34	26.79	<.0001*
200Pr,400 mM	NPr,200 mM	16.96	2.86	7.23	26.69	<.0001*
400Pr,CK	NPr,CK	15.40	2.86	5.67	25.12	<.0001*
200Pr,CK	NPr,CK	14.53	2.86	4.80	24.26	0.0002*
WPr,CK	NPr,200 mM	13.58	2.86	3.85	23.31	0.0008*
400Pr,CK	WPr,200 mM	13.48	2.86	3.75	23.21	0.0008*
WPr,400 mM	NPr,200 mM	12.90	2.86	3.17	22.62	0.0017*
200Pr,CK	WPr,200 mM	12.61	2.86	2.88	22.34	0.0024*
200Pr,200 mM	NPr,CK	12.21	2.86	2.48	21.94	0.0038*
400Pr,CK	WPr,400 mM	11.80	2.86	2.07	21.50	0.0060*
400Pr,200 mM	NPr,CK	11.31	2.86	1.58	21.04	0.0101*
WPr,200 mM	NPr,200 mM	11.21	2.86	1.49	20.94	0.0113*
400Pr,CK	WPr,CK	11.11	2.86	1.38	20.84	0.0125*
200Pr,CK	WPr,400 mM	10.93	2.86	1.20	20.66	0.0152*
200Pr,200 mM	WPr,200 mM	10.30	2.86	0.57	20.02	0.0289*

Level	- Level	Difference	Std Err Dif	Lower CL	Upper CL	p-Value
200Pr,CK	WPr,CK	10.25	2.86	0.53	19.97	0.0304*
400Pr,200 mM	WPr,200 mM	9.40	2.86	-0.33	19.12	0.06
NPr,CK	NPr,200 mM	9.30	2.86	-0.43	19.02	0.07
200Pr,200 mM	WPr,400 mM	8.61	2.86	-1.11	18.34	0.13
400Pr,400 mM	NPr,CK	8.05	2.86	-1.67	17.77	0.20
200Pr,200 mM	WPr,CK	7.93	2.86	-1.80	17.66	0.21
NPr,200 mM	NPr,400 mM	7.77	2.86	-1.96	17.49	0.244
400Pr,CK	200Pr,400 mM	7.73	2.86	-1.99	17.46	0.25
400Pr,200 mM	WPr,400 mM	7.71	2.86	-2.01	17.444	0.25
200Pr,400 mM	NPr,CK	7.66	2.86	-2.06	17.39	0.26
400Pr,CK	400Pr,400 mM	7.35	2.86	-2.37	17.07	0.31
400Pr,200 mM	WPr,CK	7.03	2.86	-2.69	16.76	0.38
200Pr,CK	200Pr,400 mM	6.87	2.86	-2.86	16.59	0.42
200Pr,CK	400Pr,400 mM	6.48	2.86	-3.24	16.21	0.51
400Pr,400 mM	WPr,200 mM	6.13	2.86	-3.59	15.86	0.59
200Pr,400 mM	WPr,200 mM	5.75	2.86	-3.97	15.47	0.68
200Pr,200 mM	200Pr,400 mM	4.55	2.86	-5.17	14.27	0.90
400Pr,400 mM	WPr,400 mM	4.45	2.86	-5.27	14.17	0.91
WPr,CK	NPr,CK	4.28	2.86	-5.44	14.01	0.93
200Pr,200 mM	400Pr,400 mM	4.16	2.86	-5.56	13.89	0.94
400Pr,CK	400Pr,200 mM	4.08	2.86	-5.64	13.81	0.95
200Pr,400 mM	WPr,400 mM	4.06	2.86	-5.66	13.79	0.95
400Pr,400 mM	WPr,CK	3.77	2.86	-5.96	13.49	0.97
400Pr,200 mM	200Pr,400 mM	3.65	2.86	-6.07	13.37	0.97
WPr,400 mM	NPr,CK	3.60	2.86	-6.12	13.32	0.98
200Pr,400 mM	WPr,CK	3.38	2.86	-6.34	13.11	0.98
400Pr,200 mM	400Pr,400 mM	3.27	2.86	-6.46	12.99	0.99
200Pr,CK	400Pr,200 mM	3.21	2.86	-6.51	12.94	0.99
400Pr,CK	200Pr,200 mM	3.18	2.86	-6.54	12.91	0.99
WPr,CK	WPr,200 mM	2.37	2.86	-7.36	12.09	0.99
200Pr,CK	200Pr,200 mM	2.31	2.86	-7.41	12.04	0.99
WPr,200 mM	NPr,CK	1.91	2.86	-7.81	11.64	0.99
WPr,400 mM	WPr,200 mM	1.68	2.86	-8.04	11.41	1.00
200Pr,200 mM	400Pr,200 mM	0.90	2.86	-8.82	10.62	1.00
400Pr,CK	200Pr,CK	0.87	2.86	-8.86	10.59	1.00
WPr,CK	WPr,400 mM	0.68	2.86	-9.04	10.41	1.00
400Pr,400 mM	200Pr,400 mM	0.38	2.86	-9.34	10.11	1.00

Fm/Fv

Analysis of variance

Source	DF	Sum of Squares	Mean Square	F Ratio
Model	11	0.27	0.02	3.41
Error	48	0.34	0.007	**Prob > F**
C. Total	59	0.61		0.0015*

Seed priming Tukey HSD

Level	- Level	Difference	Std Err Dif	Lower CL	Upper CL	p-Value
400 mM	WPr	0.05	0.031	-0.032	0.13	0.38
NP	WPr	0.05	0.031	-0.034	0.13	0.42
400 mM	200 mM	0.04	0.031	-0.046	0.12	0.64
NP	200 mM	0.03	0.031	-0.048	0.11	0.68
200 mM	WPr	0.01	0.031	-0.069	0.09	0.97
400 mM	NP	0.001	0.031	-0.0804	0.08	0.99

Treatment Tukey HSD

Level	- Level	Difference	Std Err Dif	Lower CL	Upper CL	p-Value
400 mM	CK	0.10	0.03	0.003	0.20	0.04*
400 mM	200 mM	0.07	0.03	-0.024	0.17	0.15
200 mM	CK	0.03	0.03	-0.070	0.12	0.73

Seed priming * Treatment Tukey HSD

Level	- Level	Difference	Std Err Dif	Lower CL	Upper CL	p-Value
WPr,200 mM	WPr,CK	0.24	0.05	0.06	0.43	0.0016*
NP,400 mM	WPr,CK	0.23	0.05	0.05	0.42	0.0032*
400 mM,400 mM	WPr,CK	0.23	0.05	0.04	0.41	0.0038*
400 mM,200 mM	WPr,CK	0.23	0.05	0.04	0.41	0.0049*
200 mM,400 mM	WPr,CK	0.22	0.05	0.04	0.41	0.0057*
WPr,400 mM	WPr,CK	0.21	0.05	0.03	0.39	0.0113*
NP,200 mM	WPr,CK	0.20	0.05	0.02	0.39	0.0194*
NP,CK	WPr,CK	0.16	0.05	-0.02	0.35	0.12
200 mM,200 mM	WPr,CK	0.15	0.05	-0.03	0.33	0.20
400 mM,CK	WPr,CK	0.15	0.05	-0.04	0.33	0.23
WPr,200 mM	200 mM,CK	0.12	0.05	-0.06	0.31	0.48
200 mM,CK	WPr,CK	0.12	0.05	-0.06	0.31	0.49
NP,400 mM	200 mM,CK	0.11	0.05	-0.07	0.29	0.63
400 mM,400 mM	200 mM,CK	0.11	0.05	-0.07	0.29	0.67
400 mM,200 mM	200 mM,CK	0.10	0.05	-0.08	0.29	0.72
200 mM,400 mM	200 mM,CK	0.10	0.05	-0.08	0.28	0.75
WPr,200 mM	400 mM,CK	0.10	0.05	-0.08	0.28	0.78
WPr,200 mM	200 mM,200 mM	0.09	0.05	-0.09	0.27	0.81
WPr,400 mM	200 mM,CK	0.09	0.05	-0.09	0.27	0.87
NP,400 mM	400 mM,CK	0.09	0.05	-0.09	0.27	0.88
400 mM,400 mM	400 mM,CK	0.08	0.05	-0.10	0.26	0.91
NP,400 mM	200 mM,200 mM	0.08	0.05	-0.10	0.27	0.91
WPr,200 mM	NP,CK	0.08	0.05	-0.10	0.26	0.92
400 mM,400 mM	200 mM,200 mM	0.08	0.05	-0.10	0.26	0.93
400 mM,200 mM	400 mM,CK	0.08	0.05	-0.10	0.26	0.93
NP,200 mM	200 mM,CK	0.08	0.05	-0.10	0.26	0.93
200 mM,400 mM	400 mM,CK	0.07	0.05	-0.10	0.26	0.95
400 mM,200 mM	200 mM,200 mM	0.07	0.05	-0.10	0.26	0.95

Level	- Level	Difference	Std Err Dif	Lower CL	Upper CL	p-Value
200 mM,400 mM	200 mM,200 mM	0.07	0.05	-0.11	0.26	0.96
NP,400 mM	NP,CK	0.07	0.05	-0.11	0.25	0.97
400 mM,400 mM	NP,CK	0.06	0.05	-0.11	0.25	0.98
WPr,400 mM	400 mM,CK	0.06	0.05	-0.12	0.25	0.98
400 mM,200 mM	NP,CK	0.06	0.05	-0.12	0.25	0.99
WPr,400 mM	200 mM,200 mM	0.06	0.05	-0.12	0.24	0.99
200 mM,400 mM	NP,CK	0.06	0.05	-0.13	0.24	0.99
NP,200 mM	400 mM,CK	0.05	0.05	-0.13	0.24	0.99
NP,200 mM	200 mM,200 mM	0.05	0.05	-0.13	0.23	0.99
WPr,400 mM	NP,CK	0.04	0.05	-0.13	0.23	0.99
WPr,200 mM	NP,200 mM	0.04	0.05	-0.14	0.23	0.99
NP,CK	200 mM,CK	0.04	0.05	-0.14	0.22	0.99
NP,200 mM	NP,CK	0.03	0.05	-0.14	0.22	0.99
WPr,200 mM	WPr,400 mM	0.03	0.05	-0.15	0.21	1.00
NP,400 mM	NP,200 mM	0.03	0.05	-0.15	0.21	1.00
400 mM,400 mM	NP,200 mM	0.03	0.05	-0.15	0.21	1.00
200 mM,200 mM	200 mM,CK	0.03	0.05	-0.15	0.21	1.00
400 mM,200 mM	NP,200 mM	0.02	0.05	-0.16	0.21	1.00
400 mM,CK	200 mM,CK	0.02	0.05	-0.16	0.21	1.00
200 mM,400 mM	NP,200 mM	0.02	0.05	-0.16	0.20	1.00
NP,400 mM	WPr,400 mM	0.02	0.05	-0.16	0.20	1.00
WPr,200 mM	200 mM,400 mM	0.02	0.05	-0.16	0.20	1.00
400 mM,400 mM	WPr,400 mM	0.02	0.05	-0.16	0.20	1.00
WPr,200 mM	400 mM,200 mM	0.02	0.05	-0.16	0.21	1.00
NP,CK	400 mM,CK	0.01	0.05	-0.17	0.20	1.00
400 mM,200 mM	WPr,400 mM	0.01	0.05	-0.17	0.20	1.00
WPr,200 mM	400 mM,400 mM	0.01	0.05	-0.17	0.20	1.00
NP,CK	200 mM,200 mM	0.01	0.05	-0.17	0.19	1.00
200 mM,400 mM	WPr,400 mM	0.01	0.05	-0.17	0.19	1.00
WPr,200 mM	NP,400 mM	0.01	0.05	-0.17	0.19	1.00
WPr,400 mM	NP,200 mM	0.01	0.05	-0.17	0.19	1.00
NP,400 mM	200 mM,400 mM	0.01	0.05	-0.17	0.19	1.00
NP,400 mM	400 mM,200 mM	0.007	0.05	-0.17	0.19	1.00
400 mM,400 mM	200 mM,400 mM	0.007	0.05	-0.17	0.19	1.00
400 mM,400 mM	400 mM,200 mM	0.004	0.05	-0.18	0.18	1.00
200 mM,200 mM	400 mM,CK	0.003	0.05	-0.18	0.18	1.00
NP,400 mM	400 mM,400 mM	0.003	0.05	-0.18	0.18	1.00
400 mM,200 mM	200 mM,400 mM	0.002	0.05	-0.18	0.18	1.00

μg Na^+/ g DW

Analysis of variance

Source	DF	Sum of Squares	Mean Square	F Ratio
Model	11	6922043681	629276698	11.39
Error	24	1326022951	55250956	**Prob > F**
C. Total	35	8248066632		<.0001*

Seed priming Tukey HSD

Level	- Level	Difference	Std Err Dif	Lower CL	Upper CL	p-Value
NPr	400Pr	22106.56	3503.99	12440.4	31772.72	<.0001*
NPr	200Pr	18576.67	3503.99	8910.5	28242.83	0.0001*
NPr	WPr	13139.00	3503.99	3472.8	22805.16	0.0051*
WPr	400Pr	8967.56	3503.99	-698.6	18633.72	0.07
WPr	200Pr	5437.67	3503.99	-4228.5	15103.83	0.42
200Pr	400Pr	3529.89	3503.99	-6136.3	13196.05	0.74

Treatment Tukey HSD

Level	- Level	Difference	Std Err Dif	Lower CL	Upper CL	p-Value
200 mM	CK	12681.08	3034.55	5102.94	20259.23	0.0009*
400 mM	CK	8892.50	3034.55	1314.36	16470.64	0.0193*
200 mM	400 mM	3788.58	3034.55	-3789.56	11366.73	0.43

Seed priming * Treatment Tukey HSD

Level	- Level	Difference	Std Err Dif	Lower CL	Upper CL	p-Value
NPr,200 mM	NPr,CK	50501.67	6069.10	28618.7	72384.60	<.0001*
NPr,200 mM	400Pr,400 mM	47103.33	6069.10	25220.4	68986.27	<.0001*
NPr,200 mM	400Pr,200 mM	45865.33	6069.10	23982.4	67748.27	<.0001*
NPr,200 mM	200Pr,400 mM	44997.33	6069.10	23114.4	66880.27	<.0001*
NPr,200 mM	200Pr,CK	41205.67	6069.10	19322.7	63088.60	<.0001*
NPr,200 mM	400Pr,CK	40988.00	6069.10	19105.1	62870.94	<.0001*
NPr,200 mM	WPr,CK	40074.33	6069.10	18191.4	61957.27	<.0001*
NPr,200 mM	WPr,200 mM	39016.00	6069.10	17133.1	60898.94	<.0001*
NPr,200 mM	200Pr,200 mM	37164.00	6069.10	15281.1	59046.94	0.0001*
NPr,400 mM	NPr,CK	33366.33	6069.10	11483.4	55249.27	0.0006*
NPr,400 mM	400Pr,400 mM	29968.00	6069.10	8085.1	51850.94	0.0023*
NPr,400 mM	400Pr,200 mM	28730.00	6069.10	6847.1	50612.94	0.0037*
NPr,200 mM	WPr,400 mM	27963.67	6069.10	6080.7	49846.60	0.0050*
NPr,400 mM	200Pr,400 mM	27862.00	6069.10	5979.1	49744.94	0.0052*
NPr,400 mM	200Pr,CK	24070.33	6069.10	2187.4	45953.27	0.0225*
NPr,400 mM	400Pr,CK	23852.67	6069.10	1969.7	45735.60	0.0244*
NPr,400 mM	WPr,CK	22939.00	6069.10	1056.1	44821.94	0.0342*
WPr,400 mM	NPr,CK	22538.00	6069.10	655.1	44420.94	0.0395*
NPr,400 mM	WPr,200 mM	21880.67	6069.10	-2.3	43763.60	0.05
NPr,400 mM	200Pr,200 mM	20028.67	6069.10	-1854.3	41911.60	0.09
WPr,400 mM	400Pr,400 mM	19139.67	6069.10	-2743.3	41022.60	0.13
WPr,400 mM	400Pr,200 mM	17901.67	6069.10	-3981.3	39784.60	0.18
NPr,200 mM	NPr,400 mM	17135.33	6069.10	-4747.6	39018.27	0.23
WPr,400 mM	200Pr,400 mM	17033.67	6069.10	-4849.3	38916.60	0.24
200Pr,200 mM	NPr,CK	13337.67	6069.10	-8545.3	35220.60	0.56
WPr,400 mM	200Pr,CK	13242.00	6069.10	-8640.9	35124.94	0.57
WPr,400 mM	400Pr,CK	13024.33	6069.10	-8858.6	34907.27	0.59

Level	- Level	Difference	Std Err Dif	Lower CL	Upper CL	p-Value
WPr,400 mM	WPr,CK	12110.67	6069.10	-9772.3	33993.60	0.69
WPr,200 mM	NPr,CK	11485.67	6069.10	-10397.3	33368.60	0.75
WPr,400 mM	WPr,200 mM	11052.33	6069.10	-10830.6	32935.27	0.79
NPr,400 mM	WPr,400 mM	10828.33	6069.10	-11054.6	32711.27	0.81
WPr,CK	NPr,CK	10427.33	6069.10	-11455.6	32310.27	0.84
200Pr,200 mM	400Pr,400 mM	9939.33	6069.10	-11943.6	31822.27	0.87
400Pr,CK	NPr,CK	9513.67	6069.10	-12369.3	31396.60	0.90
200Pr,CK	NPr,CK	9296.00	6069.10	-12586.9	31178.94	0.91
WPr,400 mM	200Pr,200 mM	9200.33	6069.10	-12682.6	31083.27	0.92
200Pr,200 mM	400Pr,200 mM	8701.33	6069.10	-13181.6	30584.27	0.94
WPr,200 mM	400Pr,400 mM	8087.33	6069.10	-13795.6	29970.27	0.96
200Pr,200 mM	200Pr,400 mM	7833.33	6069.10	-14049.6	29716.27	0.97
WPr,CK	400Pr,400 mM	7029.00	6069.10	-14853.9	28911.94	0.98
WPr,200 mM	400Pr,200 mM	6849.33	6069.10	-15033.6	28732.27	0.99
400Pr,CK	400Pr,400 mM	6115.33	6069.10	-15767.6	27998.27	0.99
WPr,200 mM	200Pr,400 mM	5981.33	6069.10	-15901.6	27864.27	0.99
200Pr,CK	400Pr,400 mM	5897.67	6069.10	-15985.3	27780.60	0.99
WPr,CK	400Pr,200 mM	5791.00	6069.10	-16091.9	27673.94	0.99
200Pr,400 mM	NPr,CK	5504.33	6069.10	-16378.6	27387.27	0.99
WPr,CK	200Pr,400 mM	4923.00	6069.10	-16959.9	26805.94	0.99
400Pr,CK	400Pr,200 mM	4877.33	6069.10	-17005.6	26760.27	0.99
200Pr,CK	400Pr,200 mM	4659.67	6069.10	-17223.3	26542.60	0.99
400Pr,200 mM	NPr,CK	4636.33	6069.10	-17246.6	26519.27	0.99
200Pr,200 mM	200Pr,CK	4041.67	6069.10	-17841.3	25924.60	0.99
400Pr,CK	200Pr,400 mM	4009.33	6069.10	-17873.6	25892.27	0.99
200Pr,200 mM	400Pr,CK	3824.00	6069.10	-18058.9	25706.94	0.99
200Pr,CK	200Pr,400 mM	3791.67	6069.10	-18091.3	25674.60	1.00
400Pr,400 mM	NPr,CK	3398.33	6069.10	-18484.6	25281.27	1.00
200Pr,200 mM	WPr,CK	2910.33	6069.10	-18972.6	24793.27	1.00
WPr,200 mM	200Pr,CK	2189.67	6069.10	-19693.3	24072.60	1.00
200Pr,400 mM	400Pr,400 mM	2106.00	6069.10	-19776.9	23988.94	1.00
WPr,200 mM	400Pr,CK	1972.00	6069.10	-19910.9	23854.94	1.00
200Pr,200 mM	WPr,200 mM	1852.00	6069.10	-20030.9	23734.94	1.00
400Pr,200 mM	400Pr,400 mM	1238.00	6069.10	-20644.9	23120.94	1.00
WPr,CK	200Pr,CK	1131.33	6069.10	-20751.6	23014.27	1.00
WPr,200 mM	WPr,CK	1058.33	6069.10	-20824.6	22941.27	1.00
WPr,CK	400Pr,CK	913.67	6069.10	-20969.3	22796.60	1.00
200Pr,400 mM	400Pr,200 mM	868.00	6069.10	-21014.9	22750.94	1.00
400Pr,CK	200Pr,CK	217.67	6069.10	-21665.3	22100.60	1.00

µg K^+/ g DW

Analysis of variance

Source	DF	Sum of Squares	Mean Square	F Ratio
Model	11	1.1976e+10	1.0887e+9	70.61
Error	24	370038243	15418260	**Prob > F**
C. Total	35	1.2346e+10		<.0001*

Seed priming Tukey HSD

Level	- Level	Difference	Std Err Dif	Lower CL	Upper CL	p-Value
400Pr	NPr	36504.11	1851.02	31397.9	41610.36	<.0001*
400Pr	WPr	34035.33	1851.02	28929.1	39141.58	<.0001*
200Pr	NPr	30536.22	1851.02	25430.0	35642.47	<.0001*
200Pr	WPr	28067.44	1851.02	22961.2	33173.69	<.0001*
400Pr	200Pr	5967.89	1851.02	861.6	11074.14	0.0178*
WPr	NPr	2468.78	1851.02	-2637.5	7575.02	0.55

Treatment Tukey HSD

Level	- Level	Difference	Std Err Dif	Lower CL	Upper CL	p-Value
CK	200 mM	12962.67	1603.03	8959.44	16965.90	<.0001*
CK	400 mM	10173.83	1603.03	6170.60	14177.06	<.0001*
400 mM	200 mM	2788.83	1603.03	-1214.40	6792.06	0.21

Seed priming * Treatment Tukey HSD

Level	- Level	Difference	Std Err Dif	Lower CL	Upper CL	p-Value
400Pr,CK	NPr,CK	58653.67	3206.06	47093.8	70213.55	<.0001*
400Pr,CK	NPr,200 mM	56867.67	3206.06	45307.8	68427.55	<.0001*
400Pr,CK	WPr,400 mM	56511.33	3206.06	44951.5	68071.22	<.0001*
400Pr,CK	WPr,200 mM	56468.33	3206.06	44908.5	68028.22	<.0001*
400Pr,CK	NPr,400 mM	50172.00	3206.06	38612.1	61731.88	<.0001*
400Pr,CK	WPr,CK	45307.33	3206.06	33747.5	56867.22	<.0001*
200Pr,CK	NPr,CK	42048.00	3206.06	30488.1	53607.88	<.0001*
200Pr,CK	NPr,200 mM	40262.00	3206.06	28702.1	51821.88	<.0001*
200Pr,CK	WPr,400 mM	39905.67	3206.06	28345.8	51465.55	<.0001*
200Pr,CK	WPr,200 mM	39862.67	3206.06	28302.8	51422.55	<.0001*
200Pr,400 mM	NPr,CK	34565.00	3206.06	23005.1	46124.88	<.0001*
200Pr,CK	NPr,400 mM	33566.33	3206.06	22006.5	45126.22	<.0001*
400Pr,CK	200Pr,200 mM	33390.33	3206.06	21830.5	44950.22	<.0001*
400Pr,200 mM	NPr,CK	32962.67	3206.06	21402.8	44522.55	<.0001*
200Pr,400 mM	NPr,200 mM	32779.00	3206.06	21219.1	44338.88	<.0001*
200Pr,400 mM	WPr,400 mM	32422.67	3206.06	20862.8	43982.55	<.0001*
200Pr,400 mM	WPr,200 mM	32379.67	3206.06	20819.8	43939.55	<.0001*
400Pr,200 mM	NPr,200 mM	31176.67	3206.06	19616.8	42736.55	<.0001*
400Pr,200 mM	WPr,400 mM	30820.33	3206.06	19260.5	42380.22	<.0001*
400Pr,200 mM	WPr,200 mM	30777.33	3206.06	19217.5	42337.22	<.0001*
400Pr,CK	400Pr,400 mM	30490.00	3206.06	18930.1	42049.88	<.0001*
200Pr,CK	WPr,CK	28701.67	3206.06	17141.8	40261.55	<.0001*
400Pr,400 mM	NPr,CK	28163.67	3206.06	16603.8	39723.55	<.0001*
400Pr,400 mM	NPr,200 mM	26377.67	3206.06	14817.8	37937.55	<.0001*
200Pr,400 mM	NPr,400 mM	26083.33	3206.06	14523.5	37643.22	<.0001*
400Pr,400 mM	WPr,400 mM	26021.33	3206.06	14461.5	37581.22	<.0001*
400Pr,400 mM	WPr,200 mM	25978.33	3206.06	14418.5	37538.22	<.0001*

Level	- Level	Difference	Std Err Dif	Lower CL	Upper CL	p-Value
400Pr,CK	400Pr,200 mM	25691.00	3206.06	14131.1	37250.88	<.0001*
200Pr,200 mM	NPr,CK	25263.33	3206.06	13703.5	36823.22	<.0001*
400Pr,200 mM	NPr,400 mM	24481.00	3206.06	12921.1	36040.88	<.0001*
400Pr,CK	200Pr,400 mM	24088.67	3206.06	12528.8	35648.55	<.0001*
200Pr,200 mM	NPr,200 mM	23477.33	3206.06	11917.5	35037.22	<.0001*
200Pr,200 mM	WPr,400 mM	23121.00	3206.06	11561.1	34680.88	<.0001*
200Pr,200 mM	WPr,200 mM	23078.00	3206.06	11518.1	34637.88	<.0001*
200Pr,400 mM	WPr,CK	21218.67	3206.06	9658.8	32778.55	<.0001*
400Pr,400 mM	NPr,400 mM	19682.00	3206.06	8122.1	31241.88	0.0001*
400Pr,200 mM	WPr,CK	19616.33	3206.06	8056.5	31176.22	0.0001*
200Pr,CK	200Pr,200 mM	16784.67	3206.06	5224.8	28344.55	0.0011*
200Pr,200 mM	NPr,400 mM	16781.67	3206.06	5221.8	28341.55	0.0011*
400Pr,CK	200Pr,CK	16605.67	3206.06	5045.8	28165.55	0.0013*
400Pr,400 mM	WPr,CK	14817.33	3206.06	3257.5	26377.22	0.0049*
200Pr,CK	400Pr,400 mM	13884.33	3206.06	2324.5	25444.22	0.0097*
WPr,CK	NPr,CK	13346.33	3206.06	1786.5	24906.22	0.0143*
200Pr,200 mM	WPr,CK	11917.00	3206.06	357.1	23476.88	0.0392*
WPr,CK	NPr,200 mM	11560.33	3206.06	0.45	23120.22	0.0500*
WPr,CK	WPr,400 mM	11204.00	3206.06	-355.9	22763.88	0.06
WPr,CK	WPr,200 mM	11161.00	3206.06	-398.9	22720.88	0.06
200Pr,400 mM	200Pr,200 mM	9301.67	3206.06	-2258.2	20861.55	0.20
200Pr,CK	400Pr,200 mM	9085.33	3206.06	-2474.5	20645.22	0.23
NPr,400 mM	NPr,CK	8481.67	3206.06	-3078.2	20041.55	0.31
400Pr,200 mM	200Pr,200 mM	7699.33	3206.06	-3860.5	19259.22	0.44
200Pr,CK	200Pr,400 mM	7483.00	3206.06	-4076.9	19042.88	0.48
NPr,400 mM	NPr,200 mM	6695.67	3206.06	-4864.2	18255.55	0.63
200Pr,400 mM	400Pr,400 mM	6401.33	3206.06	-5158.5	17961.22	0.69
NPr,400 mM	WPr,400 mM	6339.33	3206.06	-5220.5	17899.22	0.70
NPr,400 mM	WPr,200 mM	6296.33	3206.06	-5263.5	17856.22	0.71
WPr,CK	NPr,400 mM	4864.67	3206.06	-6695.2	16424.55	0.92
400Pr,200 mM	400Pr,400 mM	4799.00	3206.06	-6760.9	16358.88	0.93
400Pr,400 mM	200Pr,200 mM	2900.33	3206.06	-8659.5	14460.22	0.99
WPr,200 mM	NPr,CK	2185.33	3206.06	-9374.5	13745.22	0.99
WPr,400 mM	NPr,CK	2142.33	3206.06	-9417.5	13702.22	0.99
NPr,200 mM	NPr,CK	1786.00	3206.06	-9773.9	13345.88	1.00
200Pr,400 mM	400Pr,200 mM	1602.33	3206.06	-9957.5	13162.22	1.00
WPr,200 mM	NPr,200 mM	399.33	3206.06	-11160.5	11959.22	1.00
WPr,400 mM	NPr,200 mM	356.33	3206.06	-11203.5	11916.22	1.00
WPr,200 mM	WPr,400 mM	43.00	3206.06	-11516.9	11602.88	1.00

Red values mean p value< 0.05; Orange values mean p value< 0.01

Na^+ / K^+ Ratio

Analysis of variance

Source	DF	Sum of Squares	Mean Square	F Ratio
Model	11	28.71	2.61	12.13
Error	24	5.16	0.21	**Prob > F**
C. Total	35	33.87		<.0001*

Seed priming Tukey HSD

Level	- Level	Difference	Std Err Dif	Lower CL	Upper CL	p-Value
NPr	WPr	1.77	0.22	1.164	2.37	<.0001*
400Pr	WPr	1.50	0.22	0.89	2.09	<.0001*
200Pr	WPr	1.02	0.22	0.42	1.63	0.0005*
NPr	200Pr	0.74	0.22	0.14	1.35	0.0114*
400Pr	200Pr	0.47	0.22	-0.13	1.07	0.16
NPr	400Pr	0.27	0.22	-0.33	0.88	0.59

Treatment Tukey HSD

Level	- Level	Difference	Std Err Dif	Lower CL	Upper CL	p-Value
200 mM	400 mM	0.32	0.19	-0.16	0.78	0.24
200 mM	CK	0.16	0.19	-0.31	0.64	0.66
CK	400 mM	0.15	0.19	-0.32	0.62	0.71

Seed priming * Treatment Tukey HSD

Level	- Level	Difference	Std Err Dif	Lower CL	Upper CL	p-Value
NPr,200 mM	WPr,400 mM	3.40	0.38	2.03	4.76	<.0001*
NPr,200 mM	WPr,200 mM	3.24	0.38	1.88	4.60	<.0001*
NPr,200 mM	WPr,CK	2.88	0.38	1.51	4.24	<.0001*
NPr,200 mM	200Pr,200 mM	2.62	0.38	1.25	3.98	<.0001*
NPr,200 mM	NPr,CK	2.73	0.38	1.11	3.83	<.0001*
400Pr,CK	WPr,400 mM	2.25	0.38	0.89	3.62	0.0002*
400Pr,CK	WPr,200 mM	2.09	0.38	0.73	3.46	0.0005*
NPr,200 mM	400Pr,400 mM	1.98	0.38	0.61	3.35	0.0011*
NPr,200 mM	200Pr,CK	1.93	0.38	0.57	3.30	0.0015*
NPr,200 mM	400Pr,200 mM	1.94	0.38	0.55	3.30	0.0017*
NPr,200 mM	200Pr,400 mM	1.90	0.38	0.54	3.30	0.0018*
NPr,200 mM	NPr,400 mM	1.74	0.38	0.38	3.11	0.0050*
400Pr,CK	WPr,CK	1.73	0.38	0.37	3.11	0.0054*
NPr,400 mM	WPr,400 mM	1.65	0.38	0.29	3.02	0.0088*
NPr,400 mM	WPr,200 mM	1.50	0.38	0.13	2.87	0.0231*
200Pr,400 mM	WPr,400 mM	1.50	0.38	0.13	2.86	0.0231*
400Pr,200 mM	WPr,400 mM	1.50	0.38	0.12	2.85	0.0247*
400Pr,CK	200Pr,200 mM	1.47	0.38	0.11	2.83	0.0269*
200Pr,CK	WPr,400 mM	1.46	0.38	0.10	2.83	0.0278*
400Pr,400 mM	WPr,400 mM	1.42	0.38	0.05	2.78	0.0369*
200Pr,400 mM	WPr,200 mM	1.34	0.38	-0.03	2.70	0.05
400Pr,200 mM	WPr,200 mM	1.33	0.38	-0.04	2.70	0.06
400Pr,CK	NPr,CK	1.33	0.38	-0.04	2.69	0.06
200Pr,CK	WPr,200 mM	1.31	0.38	-0.05	2.67	0.07
400Pr,400 mM	WPr,200 mM	1.26	0.38	-0.10	2.62	0.09
NPr,200 mM	400Pr,CK	1.15	0.38	-0.21	2.51	0.16
NPr,400 mM	WPr,CK	1.13	0.38	-0.22	2.50	0.17

Level	- Level	Difference	Std Err Dif	Lower CL	Upper CL	p-Value
200Pr,400 mM	WPr,CK	0.97	0.38	-0.39	2.34	0.34
400Pr,200 mM	WPr,CK	0.96	0.38	-0.39	2.33	0.35
200Pr,CK	WPr,CK	0.94	0.38	-0.42	2.31	0.38
NPr,CK	WPr,400 mM	0.92	0.38	-0.47	2.29	0.41
400Pr,400 mM	WPr,CK	0.89	0.38	-0.46	2.26	0.45
NPr,400 mM	200Pr,200 mM	0.87	0.38	-0.49	2.24	0.49
400Pr,CK	400Pr,400 mM	0.83	0.38	-0.53	2.20	0.56
400Pr,CK	200Pr,CK	0.78	0.38	-0.58	2.15	0.64
200Pr,200 mM	WPr,400 mM	0.78	0.38	-0.58	2.14	0.65
NPr,CK	WPr,200 mM	0.76	0.38	-0.59	2.13	0.67
400Pr,CK	400Pr,200 mM	0.76	0.38	-0.60	2.13	0.67
400Pr,CK	200Pr,400 mM	0.75	0.38	-0.61	2.12	0.69
NPr,400 mM	NPr,CK	0.73	0.38	-0.63	2.09	0.73
200Pr,400 mM	200Pr,200 mM	0.72	0.38	-0.65	2.08	0.75
400Pr,200 mM	200Pr,200 mM	0.70	0.38	-0.66	2.07	0.77
200Pr,CK	200Pr,200 mM	0.68	0.38	-0.68	2.05	0.79
400Pr,400 mM	200Pr,200 mM	0.64	0.38	-0.73	2.00	0.86
200Pr,200 mM	WPr,200 mM	0.62	0.38	-0.74	1.98	0.87
400Pr,CK	NPr,400 mM	0.59	0.38	-0.77	1.96	0.90
200Pr,400 mM	NPr,CK	0.57	0.38	-0.79	1.93	0.92
400Pr,200 mM	NPr,CK	0.56	0.38	-0.80	1.92	0.93
200Pr,CK	NPr,CK	0.54	0.38	-0.82	1.90	0.94
WPr,CK	WPr,400 mM	0.52	0.38	-0.84	1.88	0.95
400Pr,400 mM	NPr,CK	0.54	0.38	-0.87	1.85	0.97
NPr,CK	WPr,CK	0.41	0.38	-0.95	1.77	0.99
WPr,CK	WPr,200 mM	0.36	0.38	-1.00	1.72	0.99
200Pr,200 mM	WPr,CK	0.26	0.38	-1.10	1.62	0.99
NPr,400 mM	400Pr,400 mM	0.28	0.38	-1.13	1.60	0.99
NPr,400 mM	200Pr,CK	0.19	0.38	-1.17	1.55	1.00
NPr,400 mM	400Pr,200 mM	0.17	0.38	-1.19	1.53	1.00
NPr,400 mM	200Pr,400 mM	0.16	0.38	-1.20	1.52	1.00
WPr,200 mM	WPr,400 mM	0.16	0.38	-1.20	1.52	1.00
NPr,CK	200Pr,200 mM	0.14	0.38	-1.22	1.51	1.00
200Pr,400 mM	400Pr,400 mM	0.08	0.38	-1.29	1.44	1.00
400Pr,200 mM	400Pr,400 mM	0.07	0.38	-1.29	1.43	1.00
200Pr,CK	400Pr,400 mM	0.05	0.38	-1.32	1.41	1.00
200Pr,400 mM	200Pr,CK	0.03	0.38	-1.33	1.39	1.00
400Pr,200 mM	200Pr,CK	0.02	0.38	-1.34	1.38	1.00
200Pr,400 mM	400Pr,200 mM	0.01	0.38	-1.35	1.37	1.00

μmol MDA/ g FW

Analysis of variance

Source	DF	Sum of Squares	Mean Square	F Ratio
Model	11	41.67	3.79	5.55
Error	24	16.38	0.68	**Prob > F**
C. Total	35	58.06		0.0002*

Seed priming Tukey HSD

Level	- Level	Difference	Std Err Dif	Lower CL	Upper CL	p-Value
NPr	400Pr	2.64	0.39	1.57	3.72	<.0001*
WPr	400Pr	2.17	0.39	1.09	3.24	<.0001*
NPr	200Pr	1.53	0.39	0.45	2.60	0.0033*
200Pr	400Pr	1.11	0.39	0.03	2.19	0.0404*
WPr	200Pr	1.05	0.39	-0.02	2.13	0.05
NPr	WPr	0.48	0.39	-0.60	1.55	0.62

Treatment Tukey HSD

Level	- Level	Difference	Std Err Dif	Lower CL	Upper CL	p-Value
400 mM	CK	0.77	0.34	-0.07	1.61	0.07
200 mM	CK	0.49	0.34	-0.35	1.33	0.33
400 mM	200 mM	0.28	0.34	-0.56	1.12	0.68

Seed priming * Treatment Tukey HSD

Level	- Level	Difference	Std Err Dif	Lower CL	Upper CL	p-Value
NPr,400 mM	400Pr,CK	3.16	0.67	0.73	5.59	0.0042*
NPr,400 mM	400Pr,200 mM	2.96	0.67	0.53	5.39	0.0084*
WPr,400 mM	400Pr,CK	2.87	0.67	0.44	5.30	0.0116*
NPr,200 mM	400Pr,CK	2.86	0.67	0.43	5.30	0.0117*
NPr,CK	400Pr,CK	2.71	0.67	0.27	5.14	0.0201*
WPr,400 mM	400Pr,200 mM	2.67	0.67	0.24	5.10	0.0227*
NPr,200 mM	400Pr,200 mM	2.67	0.67	0.23	5.10	0.0231*
WPr,200 mM	400Pr,CK	2.66	0.67	0.22	5.09	0.0239*
NPr,400 mM	400Pr,400 mM	2.55	0.67	0.11	4.98	0.0342*
NPr,CK	400Pr,200 mM	2.51	0.67	0.08	4.94	0.0387*
WPr,200 mM	400Pr,200 mM	2.46	0.67	0.03	4.89	0.0458*
NPr,400 mM	200Pr,CK	2.33	0.67	-0.10	4.76	0.07
WPr,400 mM	400Pr,400 mM	2.30	0.67	-0.17	4.69	0.08
NPr,200 mM	400Pr,400 mM	2.26	0.67	-0.17	4.69	0.08
NPr,CK	400Pr,400 mM	2.10	0.67	-0.33	4.53	0.14
WPr,200 mM	400Pr,400 mM	2.05	0.67	-0.38	4.48	0.16
WPr,400 mM	200Pr,CK	2.04	0.67	-0.39	4.47	0.16
NPr,200 mM	200Pr,CK	2.03	0.67	-0.40	4.46	0.16
NPr,CK	200Pr,CK	1.87	0.67	-0.55	4.31	0.25
WPr,200 mM	200Pr,CK	1.82	0.67	-0.61	4.26	0.28
WPr,CK	400Pr,CK	1.78	0.67	-0.65	4.21	0.31
200Pr,400 mM	400Pr,CK	1.76	0.67	-0.67	4.20	0.32
NPr,400 mM	200Pr,200 mM	1.61	0.67	-0.82	4.04	0.45
WPr,CK	400Pr,200 mM	1.58	0.67	-0.85	4.02	0.47
200Pr,400 mM	400Pr,200 mM	1.57	0.67	-0.87	4.00	0.48
200Pr,200 mM	400Pr,CK	1.58	0.67	-0.88	3.98	0.50
NPr,400 mM	200Pr,400 mM	1.40	0.67	-1.04	3.82	0.64
NPr,400 mM	WPr,CK	1.37	0.67	-1.05	3.81	0.66

Level	- Level	Difference	Std Err Dif	Lower CL	Upper CL	p-Value
200Pr,200 mM	400Pr,200 mM	1.35	0.67	-1.08	3.78	0.68
WPr,400 mM	200Pr,200 mM	1.32	0.67	-1.11	3.75	0.71
NPr,200 mM	200Pr,200 mM	1.31	0.67	-1.11	3.75	0.71
WPr,CK	400Pr,400 mM	1.17	0.67	-1.26	3.60	0.83
NPr,CK	200Pr,200 mM	1.16	0.67	-1.27	3.59	0.84
200Pr,400 mM	400Pr,400 mM	1.15	0.67	-1.27	3.58	0.84
WPr,200 mM	200Pr,200 mM	1.10	0.67	-1.32	3.54	0.87
WPr,400 mM	200Pr,400 mM	1.10	0.67	-1.32	3.54	0.88
NPr,200 mM	200Pr,400 mM	1.10	0.67	-1.33	3.53	0.88
WPr,400 mM	WPr,CK	1.09	0.67	-1.34	3.52	0.88
NPr,200 mM	WPr,CK	1.08	0.67	-1.35	3.51	0.89
WPr,CK	200Pr,CK	0.95	0.67	-1.50	3.38	0.95
NPr,CK	200Pr,400 mM	0.94	0.67	-1.48	3.38	0.95
200Pr,200 mM	400Pr,400 mM	0.94	0.67	-1.49	3.37	0.95
200Pr,400 mM	200Pr,CK	0.93	0.67	-1.50	3.36	0.95
NPr,CK	WPr,CK	0.93	0.67	-1.50	3.36	0.96
WPr,200 mM	200Pr,400 mM	0.89	0.67	-1.54	3.32	0.97
WPr,200 mM	WPr,CK	0.87	0.67	-1.56	3.30	0.97
200Pr,CK	400Pr,CK	0.83	0.67	-1.60	3.26	0.98
200Pr,200 mM	200Pr,CK	0.72	0.67	-1.71	3.15	0.99
200Pr,CK	400Pr,200 mM	0.63	0.67	-1.78	3.06	0.99
400Pr,400 mM	400Pr,CK	0.61	0.67	-1.82	3.04	0.99
NPr,400 mM	WPr,200 mM	0.50	0.67	-1.93	2.93	0.99
NPr,400 mM	NPr,CK	0.45	0.67	-1.98	2.88	0.99
400Pr,400 mM	400Pr,200 mM	0.41	0.67	-2.02	2.84	1.00
NPr,400 mM	NPr,200 mM	0.29	0.67	-2.14	2.72	1.00
NPr,400 mM	WPr,400 mM	0.29	0.67	-2.14	2.72	1.00
WPr,CK	200Pr,200 mM	0.23	0.67	-2.20	2.66	1.00
200Pr,CK	400Pr,400 mM	0.22	0.67	-2.21	2.65	1.00
200Pr,400 mM	200Pr,200 mM	0.21	0.67	-2.21	2.65	1.00
WPr,400 mM	WPr,200 mM	0.21	0.67	-2.22	2.64	1.00
NPr,200 mM	WPr,200 mM	0.20	0.67	-2.22	2.64	1.00
400Pr,200 mM	400Pr,CK	0.19	0.67	-2.23	2.63	1.00
WPr,400 mM	NPr,CK	0.16	0.67	-2.27	2.59	1.00
NPr,200 mM	NPr,CK	0.15	0.67	-2.27	2.60	1.00
NPr,CK	WPr,200 mM	0.05	0.67	-2.38	2.48	1.00
WPr,CK	200Pr,400 mM	0.02	0.67	-2.41	2.45	1.00
WPr,400 mM	NPr,200 mM	0.004	0.67	-2.43	2.43	1.00

mmol H_2O_2/ g FW

Analysis of variance

Source	DF	Sum of Squares	Mean Square	F Ratio
Model	11	22014.35	2001.30	2.54
Error	24	18929.88	788.74	**Prob > F**
C. Total	35	40944.23		0.0274*

Seed priming Tukey HSD

Level	- Level	Difference	Std Err Dif	Lower CL	Upper CL	p-Value
WPr	NPr	34.80	13.24	-1.71	71.33	0.06
WPr	200Pr	29.03	13.24	-7.50	65.56	0.15
WPr	400Pr	27.40	13.24	-9.12	63.92	0.19
400Pr	NPr	7.40	13.24	-29.11	43.93	0.94
200Pr	NPr	5.77	13.24	-30.75	42.29	0.97
400Pr	200Pr	1.64	13.24	-34.88	38.16	0.99

Treatment Tukey HSD

Level	- Level	Difference	Std Err Dif	Lower CL	Upper CL	p-Value
CK	400 mM	35.48	11.46	6.85	64.11	0.0132*
CK	200 mM	31.98	11.46	3.35	60.61	0.0265*
200 mM	400 mM	3.50	11.46	-25.13	32.13	0.95

Seed priming * Treatment Tukey HSD

Level	- Level	Difference	Std Err Dif	Lower CL	Upper CL	p-Value
WPr,CK	NPr,200 mM	99.78	22.93	17.10	182.47	0.0092*
WPr,CK	NPr,400 mM	93.55	22.93	10.87	176.23	0.0173*
WPr,CK	400Pr,400 mM	88.70	22.93	6.02	171.38	0.0280*
WPr,CK	200Pr,CK	78.51	22.93	-4.17	161.19	0.07
WPr,CK	200Pr,200 mM	76.29	22.93	-6.38	158.97	0.09
WPr,CK	400Pr,200 mM	76.08	22.93	-6.60	158.76	0.09
WPr,CK	WPr,400 mM	75.78	22.93	-6.88	158.48	0.09
WPr,CK	200Pr,400 mM	73.74	22.93	-8.93	156.42	0.11
WPr,CK	WPr,200 mM	65.64	22.93	-17.04	148.32	0.21
WPr,CK	400Pr,CK	58.85	22.93	-23.83	141.53	0.35
WPr,CK	NPr,CK	52.51	22.93	-30.17	135.19	0.50
NPr,CK	NPr,200 mM	47.28	22.93	-35.40	129.96	0.65
NPr,CK	NPr,400 mM	41.04	22.93	-41.63	123.73	0.80
400Pr,CK	NPr,200 mM	40.94	22.93	-41.74	123.62	0.81
NPr,CK	400Pr,400 mM	36.19	22.93	-46.50	118.87	0.90
400Pr,CK	NPr,400 mM	34.70	22.93	-47.97	117.38	0.92
WPr,200 mM	NPr,200 mM	34.15	22.93	-48.53	116.83	0.93
400Pr,CK	400Pr,400 mM	29.86	22.93	-52.82	112.53	0.97
WPr,200 mM	NPr,400 mM	27.92	22.93	-54.76	110.59	0.98
200Pr,400 mM	NPr,200 mM	26.05	22.93	-56.63	108.72	0.99
NPr,CK	200Pr,CK	26.00	22.93	-56.68	108.68	0.99
WPr,400 mM	NPr,200 mM	23.99	22.93	-58.69	106.67	0.99
NPr,CK	200Pr,200 mM	23.78	22.93	-58.89	106.46	0.99
400Pr,200 mM	NPr,200 mM	23.70	22.93	-58.97	106.38	0.99
NPr,CK	400Pr,200 mM	23.57	22.93	-59.11	106.25	0.99
200Pr,200 mM	NPr,200 mM	23.50	22.93	-59.18	106.17	0.99
NPr,CK	WPr,400 mM	23.30	22.93	-59.39	105.96	0.99

Level	- Level	Difference	Std Err Dif	Lower CL	Upper CL	p-Value
WPr,200 mM	400Pr,400 mM	23.06	22.93	-59.61	105.75	0.99
200Pr,CK	NPr,200 mM	21.28	22.93	-61.40	103.96	0.99
NPr,CK	200Pr,400 mM	21.23	22.93	-61.44	103.92	0.99
200Pr,400 mM	NPr,400 mM	19.81	22.93	-62.86	102.49	0.99
400Pr,CK	200Pr,CK	19.66	22.93	-63.01	102.34	0.99
WPr,400 mM	NPr,400 mM	17.76	22.93	-64.92	100.43	0.99
400Pr,200 mM	NPr,400 mM	17.47	22.93	-65.20	100.15	0.99
400Pr,CK	200Pr,200 mM	17.44	22.93	-65.23	100.12	0.99
200Pr,200 mM	NPr,400 mM	17.26	22.93	-65.42	99.94	0.99
400Pr,CK	400Pr,200 mM	17.23	22.93	-65.44	99.91	0.99
400Pr,CK	WPr,400 mM	16.95	22.93	-65.73	99.63	0.99
200Pr,CK	NPr,400 mM	15.04	22.93	-67.63	97.72	0.99
200Pr,400 mM	400Pr,400 mM	14.96	22.93	-67.71	97.64	0.99
400Pr,CK	200Pr,400 mM	14.89	22.93	-67.78	97.57	0.99
NPr,CK	WPr,200 mM	13.13	22.93	-69.55	95.81	1.00
WPr,400 mM	400Pr,400 mM	12.91	22.93	-69.77	95.59	1.00
WPr,200 mM	200Pr,CK	12.87	22.93	-69.80	95.55	1.00
400Pr,200 mM	400Pr,400 mM	12.62	22.93	-70.05	95.30	1.00
200Pr,200 mM	400Pr,400 mM	12.41	22.93	-70.27	95.09	1.00
400Pr,400 mM	NPr,200 mM	11.08	22.93	-71.60	93.76	1.00
WPr,200 mM	200Pr,200 mM	10.65	22.93	-72.02	93.34	1.00
WPr,200 mM	400Pr,200 mM	10.44	22.93	-72.24	93.12	1.00
200Pr,CK	400Pr,400 mM	10.19	22.93	-72.49	92.87	1.00
WPr,200 mM	WPr,400 mM	10.16	22.93	-72.52	92.84	1.00
WPr,200 mM	200Pr,400 mM	8.10	22.93	-74.58	90.78	1.00
400Pr,CK	WPr,200 mM	6.79	22.93	-75.89	89.47	1.00
NPr,CK	400Pr,CK	6.33	22.93	-76.34	89.01	1.00
NPr,400 mM	NPr,200 mM	6.23	22.93	-76.45	88.91	1.00
400Pr,400 mM	NPr,400 mM	4.85	22.93	-77.83	87.53	1.00
200Pr,400 mM	200Pr,CK	4.77	22.93	-77.91	87.45	1.00
WPr,400 mM	200Pr,CK	2.71	22.93	-79.97	85.39	1.00
200Pr,400 mM	200Pr,200 mM	2.55	22.93	-80.12	85.23	1.00
400Pr,200 mM	200Pr,CK	2.43	22.93	-80.25	85.11	1.00
200Pr,400 mM	400Pr,200 mM	2.34	22.93	-80.34	85.02	1.00
200Pr,200 mM	200Pr,CK	2.21	22.93	-80.46	84.89	1.00
200Pr,400 mM	WPr,400 mM	2.05	22.93	-80.62	84.73	1.00
WPr,400 mM	200Pr,200 mM	0.49	22.93	-82.18	83.17	1.00
WPr,400 mM	400Pr,200 mM	0.28	22.93	-82.39	82.96	1.00
400Pr,200 mM	200Pr,200 mM	0.21	22.93	-82.46	82.89	1.00

TEAC (µmol Trolox equivalent / g FW)

Analysis of variance

Source	DF	Sum of Squares	Mean Square	F Ratio
Model	11	19.70	1.79	3.28
Error	24	13.09	0.54	**Prob > F**
C. Total	35	32.80		0.0072*

Seed priming Tukey HSD

Level	- Level	Difference	Std Err Dif	Lower CL	Upper CL	p-Value
NPr	400Pr	1.41	0.35	0.45	2.37	0.0024*
WPr	400Pr	1.31	0.35	0.36	2.27	0.0047*
NPr	200Pr	1.27	0.35	0.31	2.24	0.0063*
WPr	200Pr	1.18	0.35	0.22	2.14	0.0120*
200Pr	400Pr	0.13	0.35	-0.82	1.10	0.98
NPr	WPr	0.09	0.35	-0.86	1.05	0.99

Treatment Tukey HSD

Level	- Level	Difference	Std Err Dif	Lower CL	Upper CL	p-Value
CK	200 mM	0.34	0.30	-0.41	1.09	0.51
CK	400 mM	0.26	0.30	-0.49	1.01	0.66
400 mM	200 mM	0.07	0.30	-0.67	0.83	0.96

Seed priming * Treatment Tukey HSD

Level	- Level	Difference	Std Err Dif	Lower CL	Upper CL	p-Value
NPr,CK	400Pr,CK	2.59	0.60	0.41	4.76	0.0106*
WPr,CK	400Pr,CK	2.18	0.60	0.005	4.35	0.0491*
NPr,CK	200Pr,400 mM	2.08	0.60	-0.08	4.26	0.07
NPr,CK	400Pr,200 mM	2.08	0.60	-0.09	4.25	0.07
NPr,CK	200Pr,200 mM	2.04	0.60	-0.12	4.22	0.07
NPr,CK	200Pr,CK	1.82	0.60	-0.35	3.99	0.16
WPr,200 mM	400Pr,CK	1.70	0.60	-0.46	3.88	0.23
NPr,CK	400Pr,400 mM	1.69	0.60	-0.47	3.87	0.23
WPr,CK	200Pr,400 mM	1.67	0.60	-0.50	3.85	0.25
WPr,CK	400Pr,200 mM	1.67	0.60	-0.50	3.84	0.25
WPr,CK	200Pr,200 mM	1.63	0.60	-0.53	3.81	0.27
NPr,400 mM	400Pr,CK	1.62	0.60	-0.54	3.80	0.28
WPr,400 mM	400Pr,CK	1.47	0.60	-0.70	3.64	0.42
NPr,200 mM	400Pr,CK	1.42	0.60	-0.74	3.60	0.46
WPr,CK	200Pr,CK	1.41	0.60	-0.76	3.59	0.47
WPr,CK	400Pr,400 mM	1.28	0.60	-0.88	3.46	0.60
WPr,200 mM	200Pr,400 mM	1.20	0.60	-0.97	3.38	0.69
WPr,200 mM	400Pr,200 mM	1.19	0.60	-0.98	3.37	0.69
WPr,200 mM	200Pr,200 mM	1.16	0.60	-1.01	3.34	0.73
NPr,CK	NPr,200 mM	1.16	0.60	-1.01	3.34	0.73
NPr,400 mM	200Pr,400 mM	1.12	0.60	-1.05	3.30	0.76
NPr,CK	WPr,400 mM	1.12	0.60	-1.05	3.29	0.77
NPr,400 mM	400Pr,200 mM	1.11	0.60	-1.06	3.29	0.77
NPr,400 mM	200Pr,200 mM	1.08	0.60	-1.09	3.25	0.80
WPr,400 mM	200Pr,400 mM	0.96	0.60	-1.21	3.14	0.89
NPr,CK	NPr,400 mM	0.96	0.60	-1.21	3.13	0.89
WPr,400 mM	400Pr,200 mM	0.96	0.60	-1.21	3.13	0.89

Level	- Level	Difference	Std Err Dif	Lower CL	Upper CL	p-Value
WPr,200 mM	200Pr,CK	0.94	0.60	-1.23	3.11	0.90
WPr,400 mM	200Pr,200 mM	0.93	0.60	-1.25	3.10	0.91
NPr,200 mM	200Pr,400 mM	0.92	0.60	-1.25	3.09	0.91
NPr,200 mM	400Pr,200 mM	0.91	0.60	-1.26	3.09	0.92
400Pr,400 mM	400Pr,CK	0.89	0.60	-1.28	3.07	0.93
NPr,200 mM	200Pr,200 mM	0.88	0.60	-1.29	3.06	0.93
NPr,CK	WPr,200 mM	0.88	0.60	-1.29	3.06	0.93
NPr,400 mM	200Pr,CK	0.86	0.60	-1.31	3.03	0.94
WPr,200 mM	400Pr,400 mM	0.81	0.60	-1.36	2.99	0.96
200Pr,CK	400Pr,CK	0.77	0.60	-1.40	2.94	0.97
WPr,CK	NPr,200 mM	0.75	0.60	-1.42	2.93	0.97
NPr,400 mM	400Pr,400 mM	0.73	0.60	-1.44	2.97	0.98
WPr,CK	WPr,400 mM	0.71	0.60	-1.46	2.88	0.98
WPr,400 mM	200Pr,CK	0.70	0.60	-1.47	2.88	0.98
NPr,200 mM	200Pr,CK	0.66	0.60	-1.51	2.83	0.99
WPr,400 mM	400Pr,400 mM	0.57	0.60	-1.60	2.75	0.99
WPr,CK	NPr,400 mM	0.55	0.60	-1.62	2.73	0.99
200Pr,200 mM	400Pr,CK	0.54	0.60	-1.63	2.71	0.99
NPr,200 mM	400Pr,400 mM	0.53	0.60	-1.64	2.71	0.99
400Pr,200 mM	400Pr,CK	0.51	0.60	-1.66	2.68	0.99
200Pr,400 mM	400Pr,CK	0.50	0.60	-1.67	2.68	0.99
WPr,CK	WPr,200 mM	0.47	0.60	-1.70	2.64	0.99
NPr,CK	WPr,CK	0.41	0.60	-1.76	2.58	0.99
400Pr,400 mM	200Pr,400 mM	0.39	0.60	-1.78	2.56	0.99
400Pr,400 mM	400Pr,200 mM	0.38	0.60	-1.79	2.56	0.99
400Pr,400 mM	200Pr,200 mM	0.35	0.60	-1.82	2.52	1.00
WPr,200 mM	NPr,200 mM	0.28	0.60	-1.89	2.45	1.00
200Pr,CK	200Pr,400 mM	0.26	0.60	-1.91	2.43	1.00
200Pr,CK	400Pr,200 mM	0.26	0.60	-1.91	2.43	1.00
WPr,200 mM	WPr,400 mM	0.24	0.60	-1.93	2.41	1.00
200Pr,CK	200Pr,200 mM	0.22	0.60	-1.95	2.40	1.00
NPr,400 mM	NPr,200 mM	0.20	0.60	-1.97	2.37	1.00
NPr,400 mM	WPr,400 mM	0.16	0.60	-2.01	2.33	1.00
400Pr,400 mM	200Pr,CK	0.13	0.60	-2.04	2.30	1.00
WPr,200 mM	NPr,400 mM	0.08	0.60	-2.09	2.25	1.00
WPr,400 mM	NPr,200 mM	0.04	0.60	-2.13	2.22	1.00
200Pr,200 mM	200Pr,400 mM	0.04	0.60	-2.13	2.21	1.00
200Pr,200 mM	400Pr,200 mM	0.03	0.60	-2.14	2.20	1.00
400Pr,200 mM	200Pr,400 mM	0.006	0.60	-2.16	2.18	1.00

EU Super Oxide Dismutase / mg protein

Analysis of variance

Source	DF	Sum of Squares	Mean Square	F Ratio
Model	11	553.31	50.30	4.56
Error	60	661.12	11.01	**Prob > F**
C. Total	71	1214.43		<.0001*

Seed priming Tukey HSD

Level	- Level	Difference	Std Err Dif	Lower CL	Upper CL	p-Value
NPr	400Pr	0.33	1.10	-2.59	3.25	0.99
WPr	400Pr	0.17	1.10	-2.75	3.09	0.99
200Pr	400Pr	0.17	1.10	-2.75	3.09	0.99
NPr	WPr	0.15	1.10	-2.77	3.07	0.99
NPr	200Pr	0.15	1.10	-2.77	3.07	0.99
200Pr	WPr	0.00	1.10	-2.92	2.92	1.00

Treatment Tukey HSD

Level	- Level	Difference	Std Err Dif	Lower CL	Upper CL	p-Value
CK	400 mM	6.16	0.94	3.86	8.47	<.0001*
200 mM	400 mM	3.49	0.94	1.18	5.79	0.0016*
CK	200 mM	2.67	0.94	0.37	4.98	0.0190*

Seed priming * Treatment Tukey HSD

Level	- Level	Difference	Std Err Dif	Lower CL	Upper CL	p-Value
400Pr,CK	400Pr,400 mM	8.99	1.91	2.47	15.51	0.0009*
400Pr,CK	WPr,400 mM	8.47	1.91	1.96	14.99	0.0023*
400Pr,CK	200Pr,400 mM	8.47	1.91	1.96	14.98	0.0023*
400Pr,CK	NPr,400 mM	8.20	1.91	1.68	14.71	0.0036*
400Pr,CK	400Pr,200 mM	7.46	1.91	0.95	13.98	0.0122*
WPr,CK	400Pr,400 mM	5.90	1.91	-0.61	12.41	0.11
200Pr,CK	400Pr,400 mM	5.90	1.91	-0.61	12.41	0.11
NPr,CK	400Pr,400 mM	5.70	1.91	-0.81	12.21	0.14
WPr,CK	200Pr,400 mM	5.38	1.91	-1.13	11.89	0.20
200Pr,CK	200Pr,400 mM	5.38	1.91	-1.13	11.89	0.20
WPr,CK	WPr,400 mM	5.38	1.91	-1.13	11.89	0.20
200Pr,CK	WPr,400 mM	5.38	1.91	-1.13	11.89	0.20
NPr,CK	200Pr,400 mM	5.18	1.91	-1.33	11.69	0.25
NPr,CK	WPr,400 mM	5.18	1.91	-1.33	11.69	0.25
WPr,CK	NPr,400 mM	5.10	1.91	-1.40	11.62	0.27
200Pr,CK	NPr,400 mM	5.10	1.91	-1.40	11.62	0.27
NPr,200 mM	400Pr,400 mM	5.01	1.91	-1.50	11.53	0.29
NPr,CK	NPr,400 mM	4.90	1.91	-1.60	11.42	0.32
WPr,200 mM	400Pr,400 mM	4.62	1.91	-1.89	11.14	0.41
200Pr,200 mM	400Pr,400 mM	4.62	1.91	-1.89	11.14	0.41
NPr,200 mM	WPr,400 mM	4.49	1.91	-2.02	11.01	0.45
NPr,200 mM	200Pr,400 mM	4.49	1.91	-2.02	11.01	0.45
WPr,CK	400Pr,200 mM	4.37	1.91	-2.14	10.88	0.50
200Pr,CK	400Pr,200 mM	4.37	1.91	-2.14	10.88	0.50
400Pr,CK	WPr,200 mM	4.36	1.91	-2.14	10.88	0.50
400Pr,CK	200Pr,200 mM	4.36	1.91	-2.14	10.88	0.50
NPr,200 mM	NPr,400 mM	4.22	1.91	-2.29	10.73	0.55

Level	- Level	Difference	Std Err Dif	Lower CL	Upper CL	p-Value
NPr,CK	400Pr,200 mM	4.17	1.92	-2.34	10.69	0.57
WPr,200 mM	WPr,400 mM	4.10	1.92	-2.41	10.62	0.59
200Pr,200 mM	WPr,400 mM	4.10	1.92	-2.41	10.62	0.59
WPr,200 mM	200Pr,400 mM	4.10	1.92	-2.41	10.62	0.59
200Pr,200 mM	200Pr,400 mM	4.10	1.92	-2.41	10.62	0.59
400Pr,CK	NPr,200 mM	3.98	1.92	-2.53	10.49	0.64
WPr,200 mM	NPr,400 mM	3.83	1.92	-2.68	10.35	0.69
200Pr,200 mM	NPr,400 mM	3.83	1.92	-2.68	10.34	0.69
NPr,200 mM	400Pr,200 mM	3.49	1.92	-3.03	10.00	0.80
400Pr,CK	NPr,CK	3.30	1.92	-3.22	9.80	0.85
WPr,200 mM	400Pr,200 mM	3.09	1.92	-3.42	9.61	0.89
200Pr,200 mM	400Pr,200 mM	3.09	1.92	-3.42	9.61	0.89
400Pr,CK	WPr,CK	3.09	1.92	-3.42	9.60	0.89
400Pr,CK	200Pr,CK	3.09	1.92	-3.42	9.60	0.89
400Pr,200 mM	400Pr,400 mM	1.53	1.92	-4.99	8.04	0.99
WPr,CK	WPr,200 mM	1.27	1.92	-5.24	7.79	0.99
200Pr,CK	WPr,200 mM	1.27	1.92	-5.24	7.79	0.99
WPr,CK	200Pr,200 mM	1.27	1.92	-5.24	7.79	0.99
200Pr,CK	200Pr,200 mM	1.27	1.92	-5.24	7.79	0.99
NPr,CK	WPr,200 mM	1.07	1.92	-5.44	7.59	1.00
NPr,CK	200Pr,200 mM	1.07	1.92	-5.44	7.59	1.00
400Pr,200 mM	200Pr,400 mM	1.01	1.92	-5.50	7.520	1.00
400Pr,200 mM	WPr,400 mM	1.01	1.92	-5.50	7.52	1.00
WPr,CK	NPr,200 mM	0.88	1.92	-5.63	7.40	1.00
200Pr,CK	NPr,200 mM	0.88	1.92	-5.63	7.40	1.00
NPr,400 mM	400Pr,400 mM	0.79	1.92	-5.72	7.30	1.00
400Pr,200 mM	NPr,400 mM	0.73	1.92	-5.78	7.25	1.00
NPr,CK	NPr,200 mM	0.68	1.92	-5.83	7.20	1.00
WPr,400 mM	400Pr,400 mM	0.52	1.92	-5.99	7.03	1.00
200Pr,400 mM	400Pr,400 mM	0.52	1.92	-5.99	7.03	1.00
NPr,200 mM	WPr,200 mM	0.39	1.92	-6.12	6.90	1.00
NPr,200 mM	200Pr,200 mM	0.39	1.92	-6.12	6.90	1.00
NPr,400 mM	200Pr,400 mM	0.27	1.92	-6.24	6.78	1.00
NPr,400 mM	WPr,400 mM	0.27	1.92	-6.24	6.78	1.00
WPr,CK	NPr,CK	0.20	1.92	-6.32	6.71	1.00
200Pr,CK	NPr,CK	0.20	1.92	-6.32	6.71	1.00
WPr,400 mM	200Pr,400 mM	4.441e-16	1.92	-6.52	6.52	1.00
200Pr,CK	WPr,CK	0.00	1.92	-6.52	6.52	1.00
200Pr,200 mM	WPr,200 mM	0.00	1.92	-6.52	6.52	1.00

CAT (µmmol H_2O_2 . min^{-1}. mg^{-1} protein)

Analysis of variance

Source	DF	Sum of Squares	Mean Square	F Ratio
Model	11	0.055	0.005	58.34
Error	24	0.002	0.00008	**Prob > F**
C. Total	35	0.057		<.0001*

Seed priming Tukey HSD

Level	- Level	Difference	Std Err Dif	Lower CL	Upper CL	p-Value
400Pr	NPr	0.046	0.0044	0.034	0.058	<.0001*
400Pr	WPr	0.044	0.0044	0.0321	0.056	<.0001*
200Pr	NPr	0.027	0.0044	0.015	0.039	<.0001*
200Pr	WPr	0.025	0.0044	0.013	0.037	<.0001*
400Pr	200Pr	0.018	0.0044	0.006	0.030	0.0014*
WPr	NPr	0.002	0.0044	-0.009	0.014	0.96

Treatment Tukey HSD

Level	- Level	Difference	Std Err Dif	Lower CL	Upper CL	p-Value
400 mM	200 mM	0.04	0.0038	0.03	0.05	<.0001*
CK	200 mM	0.04	0.0038	0.03	0.04	<.0001*
400 mM	CK	0.005	0.0038	-0.004	0.01	0.40

Seed priming * Treatment Tukey HSD

Level	- Level	Difference	Std Err Dif	Lower CL	Upper CL	p-Value
400Pr,400 mM	200Pr,200 mM	0.12	0.007	0.09	0.15	<.0001*
200Pr,CK	200Pr,200 mM	0.12	0.007	0.09	0.14	<.0001*
400Pr,400 mM	WPr,200 mM	0.12	0.007	0.09	0.14	<.0001*
200Pr,CK	WPr,200 mM	0.11	0.007	0.08	0.14	<.0001*
400Pr,400 mM	NPr,200 mM	0.11	0.007	0.08	0.14	<.0001*
400Pr,400 mM	WPr,CK	0.11	0.007	0.08	0.13	<.0001*
200Pr,CK	NPr,200 mM	0.10	0.007	0.07	0.13	<.0001*
200Pr,CK	WPr,CK	0.10	0.007	0.07	0.13	<.0001*
400Pr,400 mM	NPr,CK	0.10	0.007	0.07	0.12	<.0001*
400Pr,400 mM	NPr,400 mM	0.09	0.007	0.07	0.12	<.0001*
400Pr,400 mM	200Pr,400 mM	0.09	0.007	0.07	0.12	<.0001*
200Pr,CK	NPr,CK	0.09	0.007	0.07	0.12	<.0001*
400Pr,400 mM	400Pr,200 mM	0.09	0.007	0.07	0.12	<.0001*
200Pr,CK	NPr,400 mM	0.09	0.007	0.06	0.12	<.0001*
200Pr,CK	200Pr,400 mM	0.09	0.007	0.06	0.12	<.0001*
200Pr,CK	400Pr,200 mM	0.09	0.007	0.06	0.12	<.0001*
400Pr,400 mM	WPr,400 mM	0.07	0.007	0.05	0.103	<.0001*
400Pr,400 mM	400Pr,CK	0.07	0.007	0.05	0.102	<.0001*
200Pr,CK	WPr,400 mM	0.07	0.007	0.04	0.098	<.0001*
200Pr,CK	400Pr,CK	0.07	0.007	0.04	0.097	<.0001*
400Pr,CK	200Pr,200 mM	0.05	0.007	0.02	0.076	<.0001*
WPr,400 mM	200Pr,200 mM	0.05	0.007	0.02	0.075	<.0001*
400Pr,CK	WPr,200 mM	0.04	0.007	0.01	0.069	0.0005*
WPr,400 mM	WPr,200 mM	0.04	0.007	0.01	0.068	0.0007*
400Pr,CK	NPr,200 mM	0.03	0.007	0.003	0.062	0.0042*
WPr,400 mM	NPr,200 mM	0.03	0.007	0.007	0.061	0.0057*
400Pr,CK	WPr,CK	0.03	0.007	0.007	0.061	0.0061*

Level	- Level	Difference	Std Err Dif	Lower CL	Upper CL	p-Value
WPr,400 mM	WPr,CK	0.03	0.007	0.006	0.060	0.0083*
400Pr,200 mM	200Pr,200 mM	0.03	0.007	0.001	0.056	0.0308*
200Pr,400 mM	200Pr,200 mM	0.03	0.007	8.0943e-6	0.054	0.0499*
NPr,400 mM	200Pr,200 mM	0.02	0.007	-0.0005	0.054	0.06
400Pr,CK	NPr,CK	0.02	0.007	-0.001	0.053	0.07
WPr,400 mM	NPr,CK	0.02	0.007	-0.002	0.052	0.09
NPr,CK	200Pr,200 mM	0.02	0.007	-0.004	0.050	0.14
400Pr,CK	NPr,400 mM	0.02	0.007	-0.005	0.049	0.17
400Pr,CK	200Pr,400 mM	0.02	0.007	-0.005	0.049	0.20
400Pr,200 mM	WPr,200 mM	0.02	0.007	-0.005	0.049	0.20
WPr,400 mM	NPr,400 mM	0.02	0.007	-0.006	0.049	0.22
WPr,400 mM	200Pr,400 mM	0.02	0.007	-0.006	0.048	0.25
400Pr,CK	400Pr,200 mM	0.02	0.007	-0.007	0.047	0.29
200Pr,400 mM	WPr,200 mM	0.02	0.007	-0.007	0.047	0.29
NPr,400 mM	WPr,200 mM	0.02	0.007	-0.007	0.047	0.33
WPr,400 mM	400Pr,200 mM	0.02	0.007	-0.008	0.046	0.35
NPr,CK	WPr,200 mM	0.01	0.007	-0.011	0.043	0.58
400Pr,200 mM	NPr,200 mM	0.01	0.007	-0.012	0.042	0.68
WPr,CK	200Pr,200 mM	0.01	0.007	-0.012	0.042	0.70
400Pr,200 mM	WPr,CK	0.01	0.007	-0.013	0.041	0.77
NPr,200 mM	200Pr,200 mM	0.01	0.007	-0.013	0.041	0.79
200Pr,400 mM	NPr,200 mM	0.01	0.007	-0.014	0.040	0.81
NPr,400 mM	NPr,200 mM	0.01	0.007	-0.014	0.040	0.84
200Pr,400 mM	WPr,CK	0.01	0.007	-0.015	0.039	0.88
NPr,400 mM	WPr,CK	0.01	0.007	-0.016	0.039	0.91
NPr,CK	NPr,200 mM	0.009	0.007	-0.017	0.036	0.97
NPr,CK	WPr,CK	0.008	0.007	-0.019	0.036	0.99
WPr,CK	WPr,200 mM	0.008	0.007	-0.019	0.035	0.99
WPr,200 mM	200Pr,200 mM	0.007	0.007	-0.020	0.034	0.99
NPr,200 mM	WPr,200 mM	0.006	0.007	-0.020	0.034	0.99
400Pr,200 mM	NPr,CK	0.005	0.007	-0.021	0.032	0.99
400Pr,400 mM	200Pr,CK	0.005	0.007	-0.022	0.032	0.99
200Pr,400 mM	NPr,CK	0.004	0.007	-0.023	0.031	1.00
NPr,400 mM	NPr,CK	0.003	0.007	-0.024	0.030	1.00
400Pr,200 mM	NPr,400 mM	0.002	0.007	-0.025	0.029	1.00
400Pr,200 mM	200Pr,400 mM	0.001	0.007	-0.025	0.028	1.00
WPr,CK	NPr,200 mM	0.001	0.007	-0.026	0.028	1.00
400Pr,CK	WPr,400 mM	0.0009	0.007	-0.026	0.028	1.00
200Pr,400 mM	NPr,400 mM	0.0006	0.007	-0.027	0.028	1.00

CmSOS1 gene expression

Analysis of variance

Source	Nparm	DF	Sum of Squares	F Ratio	Prob > F
Seed priming	2	2	3.77	23.29	<.0001*
Treatment	2	2	0.07	0.47	0.62
Seed priming*Treatment	4	4	1.48	4.56	0.0054*

Seed priming Tukey HSD

Level	- Level	Difference	Std Err Dif	Lower CL	Upper CL	p-Value
400Pr	WPr	0.77	0.11	0.49	1.06	<.0001*
400 Pr	200 Pr	0.43	0.11	0.16	0.70	0.0014*
200 Pr	WPr	0.34	0.11	0.06	0.62	0.0131*

Treatment Tukey HSD

Level	- Level	Difference	Std Err Dif	Lower CL	Upper CL	p-Value
200 mM	CK	0.10	0.11	-0.16	0.38	0.60
400 Mm	CK	0.06	0.11	-0.22	0.35	0.83
200 mM	400 mM	0.04	0.11	-0.23	0.31	0.93

Seed priming * Treatment Tukey HSD

Level	- Level	Difference	Std Err Dif	Lower CL	Upper CL	p-Value
400Pr,200 mM	WPr,200 mM	1.23	0.19	0.59	1.87	<.0001*
400Pr,400 mM	WPr,200 mM	1.09	0.20	0.42	1.77	0.0002*
400Pr,200 mM	WPr,400 mM	1.01	0.19	0.37	1.65	0.0003*
400Pr,200 mM	WPr,CK	0.93	0.19	0.29	1.56	0.0010*
400Pr,400 mM	WPr,400 mM	0.88	0.20	0.20	1.55	0.0038*
400Pr,200 mM	200Pr,400 mM	0.88	0.19	0.24	1.51	0.0020*
400Pr,400 mM	WPr,CK	0.79	0.20	0.12	1.47	0.0113*
400Pr,400 mM	200Pr,400 mM	0.74	0.20	0.07	1.41	0.0210*
400Pr,200 mM	400Pr,CK	0.70	0.19	0.07	1.34	0.0208*
400Pr,200 mM	200Pr,200 mM	0.67	0.17	0.09	1.24	0.0127*
200Pr,CK	WPr,200 mM	0.64	0.20	-0.03	1.31	0.07
400Pr,200 mM	200Pr,CK	0.59	0.19	-0.04	1.22	0.08
400Pr,400 mM	400Pr,CK	0.57	0.20	-0.09	1.24	0.14
200Pr,200 mM	WPr,200 mM	0.55	0.18	-0.05	1.17	0.09
400Pr,400 mM	200Pr,200 mM	0.54	0.18	-0.07	1.15	0.19
400Pr,CK	WPr,200 mM	0.52	0.20	-0.15	1.19	0.23
400Pr,400 mM	200Pr,CK	0.45	0.20	-0.21	1.12	0.39
200Pr,CK	WPr,400 mM	0.42	0.20	-0.24	1.09	0.49
200Pr,400 mM	WPr,200 mM	0.35	0.20	-0.32	1.02	0.71
200Pr,200 mM	WPr,400 mM	0.34	0.18	-0.27	0.95	0.65
200Pr,CK	WPr,CK	0.34	0.20	-0.33	1.01	0.75
400Pr,CK	WPr,400 mM	0.30	0.20	-0.36	0.97	0.83
WPr,CK	WPr,200 mM	0.30	0.20	-0.37	0.97	0.84
200Pr,CK	WPr,400 mM	0.29	0.20	-0.38	0.96	0.87
200Pr,200 mM	WPr,CK	0.25	0.18	-0.35	0.86	0.89
400Pr,CK	WPr,CK	0.22	0.20	-0.45	0.89	0.97
WPr,400 mM	WPr,200 mM	0.21	0.20	-0.45	0.88	0.97
200Pr,200 mM	WPr,400 mM	0.20	0.18	-0.40	0.82	0.96
400Pr,CK	WPr,400 mM	0.17	0.20	-0.50	0.84	0.99
200Pr,400 mM	WPr,400 mM	0.13	0.20	-0.53	0.80	0.99
400Pr,200 mM	400Pr,400 mM	0.13	0.19	-0.50	0.77	0.99
200Pr,CK	400Pr,CK	0.12	0.20	-0.55	0.79	0.99

Red values mean p value< 0.05; Orange values mean p value< 0.01

Level	- Level	Difference	Std Err Dif	Lower CL	Upper CL	p-Value
WPr,CK	WPr,400 mM	0.08	0.20	-0.588	0.75	1.00
200Pr,CK	WPr,200 mM	0.08	0.18	-0.53	0.69	0.99
200Pr,400 mM	WPr,CK	0.05	0.20	-0.62	0.72	1.00
200Pr,200 mM	400Pr,CK	0.034	0.18	-0.58	0.65	1.00

CmSOS2 gene expression

Analysis of variance

Source	DF	Sum of Squares	Mean Square	F Ratio
Model	8	5884.69	735.58	123.48
Error	37	220.39	5.95	**Prob > F**
C. Total	45	6105.09		<.0001*

Seed priming Tukey HSD

Level	- Level	Difference	Std Err Dif	Lower CL	Upper CL	p-Value
400Pr	200Pr	15.27	0.94	12.97	17.56	<.0001*
400Pr	WPr	15.18	0.89	13.00	17.36	<.0001*
WPr	200Pr	0.08	0.89	-2.08	2.26	0.99

Treatment Tukey HSD

Level	- Level	Difference	Std Err Dif	Lower CL	Upper CL	p-Value
200 mM	CK	10.56	0.90	8.34	12.78	<.0001*
200 mM	400 mM	6.67	0.86	4.57	8.77	<.0001*
400 mM	CK	3.89	0.95	1.57	6.21	0.0006*

Seed priming * Treatment Tukey HSD

Level	- Level	Difference	Std Err Dif	Lower CL	Upper CL	p-Value
400Pr,200 mM	200Pr,200 mM	34.02	1.409107	29.3846	38.66126	<.0001*
400Pr,200 mM	WPr,200 mM	33.47	1.409107	28.8367	38.11330	<.0001*
400Pr,200 mM	WPr,400 mM	33.33	1.477884	28.4694	38.19884	<.0001*
400Pr,200 mM	200Pr,CK	33.28	1.725797	27.6027	38.96420	<.0001*
400Pr,200 mM	WPr,CK	33.01	1.409107	28.3755	37.65217	<.0001*
400Pr,200 mM	400Pr,CK	32.87	1.575430	27.6904	38.06197	<.0001*
400Pr,200 mM	200Pr,400 mM	32.77	1.409107	28.1408	37.41741	<.0001*
400Pr,200 mM	400Pr,400 mM	21.39	1.575430	16.2061	26.57766	<.0001*
400Pr,400 mM	200Pr,200 mM	12.63	1.575430	7.4453	17.81687	<.0001*
400Pr,400 mM	WPr,200 mM	12.08	1.575430	6.8973	17.26891	<.0001*
400Pr,400 mM	WPr,400 mM	11.94	1.637235	6.5530	17.33151	<.0001*
400Pr,400 mM	200Pr,CK	11.89	1.864074	5.7557	18.02750	<.0001*
400Pr,400 mM	WPr,CK	11.62	1.575430	6.4362	16.80779	<.0001*
400Pr,400 mM	400Pr,CK	11.48	1.725797	5.8036	17.16506	<.0001*
400Pr,400 mM	200Pr,400 mM	11.38	1.575430	6.2014	16.57303	<.0001*
200Pr,400 mM	200Pr,200 mM	1.24	1.409107	-3.3945	5.88216	0.9926
400Pr,CK	200Pr,200 mM	1.14	1.575430	-4.0390	6.33257	0.9980
WPr,CK	200Pr,200 mM	1.00	1.409107	-3.6292	5.64740	0.9982
200Pr,CK	200Pr,200 mM	0.74	1.725797	-4.9413	6.42025	1.0000
200Pr,400 mM	WPr,200 mM	0.69	1.409107	-3.9424	5.33420	0.9999
WPr,400 mM	200Pr,200 mM	0.68	1.477884	-4.1759	5.55351	0.9999
400Pr,CK	WPr,200 mM	0.59	1.575430	-4.5870	5.78460	1.0000
200Pr,400 mM	WPr,400 mM	0.55	1.477884	-4.3097	5.41975	1.0000
WPr,200 mM	200Pr,200 mM	0.54	1.409107	-4.0904	5.18628	1.0000
200Pr,400 mM	200Pr,CK	0.50	1.725797	-5.1764	6.18511	1.0000
WPr,CK	WPr,200 mM	0.46	1.409107	-4.1772	5.09944	1.0000
400Pr,CK	WPr,400 mM	0.45	1.637235	-4.9313	5.84720	1.0000
400Pr,CK	200Pr,CK	0.40	1.864074	-5.7286	6.54319	1.0000
WPr,CK	WPr,400 mM	0.32	1.477884	-4.5444	5.18499	1.0000
WPr,CK	200Pr,CK	0.26	1.725797	-5.4112	5.95035	1.0000

Level	- Level	Difference	Std Err Dif	Lower CL	Upper CL	p-Value
200Pr,400 mM	WPr,CK	0.23	1.40	-4.40	4.87	1.00
200Pr,CK	WPr,200 mM	0.19	1.72	-5.49	5.87	1.00
WPr,400 mM	WPr,200 mM	0.14	1.47	-4.72	5.00	1.00
400Pr,CK	WPr,CK	0.13	1.57	-5.04	5.32	1.00
200Pr,400 mM	400Pr,CK	0.09	1.57	-5.08	5.28	1.00
200Pr,CK	WPr,400 mM	0.05	1.78	-5.81	5.91	1.00

CmNHX1 gene expression

Analysis of variance

Source	DF	Sum of Squares	Mean Square	F Ratio
Model	8	32.22	4.03	5.34
Error	36	27.16	0.75	**Prob > F**
C. Total	44	59.39		0.0002*

Seed priming Tukey HSD

Level	- Level	Difference	Std Err Dif	Lower CL	Upper CL	p-Value
400Pr	200Pr	1.17	0.32	0.37	1.96	0.0026*
400Pr	WPr	0.75	0.33	-0.05	1.55	0.07
WPr	200Pr	0.41	0.32	-0.35	1.19	0.39

Treatment Tukey HSD

Level	- Level	Difference	Std Err Dif	Lower CL	Upper CL	p-Value
400 mM	CK	1.30	0.33	0.49	2.10	0.0010*
400 mM	200 mM	1.04	0.32	0.24	1.83	0.0078*
200 mM	CK	0.26	0.31	-0.51	1.04	0.68

Seed priming * Treatment Tukey HSD

Level	- Level	Difference	Std Err Dif	Lower CL	Upper CL	p-Value
400Pr,400 mM	WPr,CK	2.94	0.587	1.023	4.86	0.0004*
400Pr,400 mM	200Pr,400 mM	2.81	0.56	0.96	4.66	0.0004*
400Pr,400 mM	200Pr,200 mM	2.59	0.56	0.75	4.44	0.0014*
400Pr,400 mM	WPr,200 mM	2.54	0.56	0.69	4.39	0.0018*
400Pr,400 mM	400Pr,CK	2.44	0.56	0.59	4.29	0.0030*
400Pr,400 mM	200Pr,CK	2.38	0.61	0.36	4.41	0.0112*
WPr,400 mM	WPr,CK	1.88	0.58	-0.04	3.80	0.05
400Pr,400 mM	400Pr,200 mM	1.84	0.61	-0.17	3.87	0.09
WPr,400 mM	200Pr,400 mM	1.75	0.56	-0.09	3.60	0.07
WPr,400 mM	200Pr,200 mM	1.53	0.56	-0.31	3.38	0.17
WPr,400 mM	WPr,200 mM	1.47	0.56	-0.37	3.32	0.20
WPr,400 mM	400Pr,CK	1.38	0.56	-0.46	3.23	0.28
WPr,400 mM	200Pr,CK	1.32	0.61	-0.70	3.34	0.45
400Pr,200 mM	WPr,CK	1.09	0.58	-0.82	3.01	0.63
400Pr,400 mM	WPr,400 mM	1.06	0.61	-0.96	3.08	0.72
400Pr,200 mM	200Pr,400 mM	0.96	0.56	-0.88	2.81	0.72
WPr,400 mM	400Pr,200 mM	0.78	0.61	-1.23	2.81	0.93
400Pr,200 mM	200Pr,200 mM	0.75	0.56	-1.09	2.59	0.91
400Pr,200 mM	WPr,200 mM	0.69	0.56	-1.15	2.54	0.94
400Pr,200 mM	400Pr,CK	0.59	0.56	-1.25	2.44	0.97
200Pr,CK	WPr,CK	0.55	0.58	-1.36	2.47	0.98
400Pr,200 mM	200Pr,CK	0.53	0.61	-1.48	2.56	0.99
400Pr,CK	WPr,CK	0.49	0.52	-1.23	2.23	0.98
200Pr,CK	200Pr,400 mM	0.42	0.56	-1.41	2.27	0.99
WPr,200 mM	WPr,CK	0.40	0.52	-1.33	2.13	0.99
400Pr,CK	200Pr,400 mM	0.36	0.50	-1.28	2.02	0.99
200Pr,200 mM	WPr,CK	0.34	0.52	-1.38	2.07	0.99
WPr,200 mM	200Pr,400 mM	0.27	0.50	-1.37	1.92	0.99
200Pr,200 mM	200Pr,400 mM	0.21	0.50	-1.43	1.86	1.00
200Pr,CK	200Pr,200 mM	0.21	0.56	-1.63	2.06	1.00
200Pr,CK	WPr,200 mM	0.15	0.56	-1.69	2.00	1.00
400Pr,CK	200Pr,200 mM	0.15	0.50	-1.50	1.80	1.00
200Pr,400 mM	WPr,CK	0.12	0.52	-1.60	1.86	1.00
400Pr,CK	WPr,200 mM	0.09	0.50	-1.55	1.74	1.00
200Pr,CK	400Pr,CK	0.06	0.56	-1.78	1.90	1.00
WPr,200 mM	200Pr,200 mM	0.05	0.50	-1.59	1.71	1.00

Red values mean p value< 0.05; Orange values mean p value< 0.01

CmWRKY25 gene expression

Analysis of variance

Source	DF	Sum of Squares	Mean Square	F Ratio
Model	8	1023.39	127.92	7.60
Error	54	908.36	16.82	**Prob > F**
C. Total	62	1931.76		<.0001*

Seed priming Tukey HSD

Level	- Level	Difference	Std Err Dif	Lower CL	Upper CL	p-Value
200Pr	WPr	4.32	1.34	1.08	7.56	0.0062*
400Pr	WPr	4.32	1.24	1.31	7.33	0.0030*
200Pr	400Pr	0.002	1.30	-3.14	3.15	1.00

Treatment Tukey HSD

Level	- Level	Difference	Std Err Dif	Lower CL	Upper CL	p-Value
400 mM	CK	5.73	1.28	2.64	8.83	0.0001*
200 mM	CK	3.10	1.33	-0.11	6.32	0.06
400 mM	200 mM	2.63	1.28	-0.45	5.72	0.10

Seed priming * Treatment Tukey HSD

Level	- Level	Difference	Std Err Dif	Lower CL	Upper CL	p-Value
400Pr,400 mM	WPr,CK	12.71	2.36	5.05	20.35	<.0001*
400Pr,400 mM	200Pr,CK	12.31	2.36	4.66	19.96	0.0001*
400Pr,400 mM	WPr,200 mM	12.19	2.48	4.16	20.21	0.0003*
400Pr,400 mM	WPr,400 mM	11.94	2.16	4.95	18.92	<.0001*
400Pr,400 mM	200Pr,400 mM	11.73	2.16	4.75	18.72	<.0001*
400Pr,400 mM	400Pr,CK	11.62	2.16	4.64	18.61	<.0001*
200Pr,200 mM	WPr,CK	9.22	2.48	1.20	17.24	0.0132*
200Pr,200 mM	200Pr,CK	8.83	2.48	0.80	16.85	0.0208*
200Pr,200 mM	WPr,200 mM	8.71	2.59	0.33	17.09	0.0358*
200Pr,200 mM	WPr,400 mM	8.46	2.28	1.07	15.85	0.0139*
200Pr,200 mM	200Pr,400 mM	8.25	2.28	0.86	15.64	0.0179*
200Pr,200 mM	400Pr,CK	8.14	2.28	0.75	15.53	0.0205*
400Pr,400 mM	400Pr,200 mM	7.99	2.21	0.84	15.15	0.0179*
400Pr,200 mM	WPr,CK	4.70	2.21	-2.44	11.86	0.46
200Pr,200 mM	400Pr,200 mM	4.51	2.33	-3.03	12.07	0.59
400Pr,200 mM	200Pr,CK	4.31	2.21	-2.83	11.47	0.58
400Pr,200 mM	WPr,200 mM	4.19	2.33	-3.35	11.75	0.68
400Pr,200 mM	WPr,400 mM	3.94	1.99	-2.49	10.38	0.56
400Pr,200 mM	200Pr,400 mM	3.73	1.99	-2.69	10.17	0.63
400Pr,200 mM	400Pr,CK	3.63	1.99	-2.80	10.06	0.66
400Pr,400 mM	200Pr,200 mM	3.48	2.48	-4.54	11.50	0.89
400Pr,CK	WPr,CK	1.07	2.16	-5.90	8.06	0.99
200Pr,400 mM	WPr,CK	0.96	2.16	-6.01	7.95	0.99
WPr,400 mM	WPr,CK	0.76	2.16	-6.22	7.74	1.00
400Pr,CK	200Pr,CK	0.68	2.16	-6.29	7.66	1.00
200Pr,400 mM	200Pr,CK	0.57	2.16	-6.40	7.56	1.00
400Pr,CK	WPr,200 mM	0.56	2.28	-6.82	7.95	1.00
WPr,200 mM	WPr,CK	0.51	2.48	-7.51	8.53	1.00
200Pr,400 mM	WPr,200 mM	0.45	2.28	-6.93	7.84	1.00
200Pr,CK	WPr,CK	0.39	2.36	-7.25	8.04	1.00
WPr,400 mM	200Pr,CK	0.37	2.16	-6.61	7.35	1.00
400Pr,CK	WPr,400 mM	0.31	1.93	-5.93	6.56	1.00
WPr,400 mM	WPr,200 mM	0.25	2.28	-7.13	7.64	1.00
200Pr,400 mM	WPr,400 mM	0.20	1.93	-6.04	6.45	1.00
WPr,200 mM	200Pr,CK	0.12	2.48	-7.90	8.14	1.00
400Pr,CK	200Pr,400 mM	0.10	1.93	-6.13	6.35	1.00

Red values mean p value< 0.05; Orange values mean p value< 0.01

Root Na^+

Analysis of variance

Source	DF	Sum of Squares	Mean Square	F Ratio
Model	8	2308.42	288.55	31.88
Error	18	162.89	9.05	Prob > F
C. Total	26	2471.32		<.0001*

Seed priming Tukey HSD

Level	- Level	Difference	Std Err Dif	Lower CL	Upper CL	p-Value
SP400	SP0	3.70	1.418	0.083	7.32	0.0445*
SP200	SP0	3.09	1.418	-0.521	6.71	0.1011
SP400	SP200	0.60	1.418	-3.014	4.22	0.9051

Treatment Tukey HSD

Level	- Level	Difference	Std Err Dif	Lower CL	Upper CL	p-Value
400mM	0mM	21.33	1.418	17.71	24.94	<.0001*
200mM	0mM	11.29	1.418	7.67	14.91	<.0001*
400mM	200mM	10.03	1.418	6.42	13.65	<.0001*

Seed priming * Treatment Tukey HSD

Level	- Level	Difference	Std Err Dif	Lower CL	Upper CL	p-Value
SP400,400mM	SP200,0mM	28.13	2.45	19.52	36.74	<.0001*
SP400,400mM	SP400,0mM	25.56	2.45	16.95	34.16	<.0001*
SP200,400mM	SP200,0mM	24.74	2.45	16.13	33.34	<.0001*
SP400,400mM	SP0,0mM	23.883	2.45	15.27	32.48	<.0001*
SP200,400mM	SP400,0mM	22.17	2.45	13.56	30.77	<.0001*
SP200,400mM	SP0,0mM	20.49	2.45	11.88	29.09	<.0001*
SP0,400mM	SP200,0mM	17.91	2.45	9.329	26.50	<.0001*
SP400,400mM	SP0,200mM	17.67	2.45	9.07	26.28	<.0001*
SP200,200mM	SP200,0mM	17.19	2.45	8.58	25.79	<.0001*
SP0,400mM	SP400,0mM	15.36	2.45	6.758	23.97	0.0002*
SP400,400mM	SP400,200mM	15.08	2.45	6.48	23.69	0.0002*
SP200,200mM	SP400,0mM	14.62	2.45	6.01	23.22	0.0003*
SP200,400mM	SP0,200mM	14.28	2.45	5.68	22.89	0.0004*
SP0,400mM	SP0,0mM	13.68	2.45	5.07	22.29	0.0007*
SP400,200mM	SP200,0mM	13.04	2.45	4.43	21.65	0.0012*
SP200,200mM	SP0,0mM	12.94	2.45	4.33	21.54	0.0013*
SP200,400mM	SP400,200mM	11.69	2.45	3.09	20.30	0.0038*
SP400,400mM	SP200,200mM	10.94	2.45	2.33	19.54	0.0073*
SP400,200mM	SP400,0mM	10.47	2.45	1.8	19.08	0.0108*
SP0,200mM	SP200,0mM	10.45	2.45	1.84	19.06	0.0109*
SP400,400mM	SP0,400mM	10.19	2.45	1.59	18.80	0.0136*
SP400,200mM	SP0,0mM	8.79	2.45	0.18	17.40	0.0430*
SP0,200mM	SP400,0mM	7.88	2.45	-0.72	16.49	0.08
SP200,400mM	SP200,200mM	7.55	2.45	-1.055	16.15	0.11
SP0,400mM	SP0,200mM	7.47	2.45	-1.12	16.08	0.11
SP200,400mM	SP0,400mM	6.80	2.45	-1.79	15.41	0.19
SP200,200mM	SP0,200mM	6.73	2.45	-1.86	15.34	0.20
SP0,200mM	SP0,0mM	6.20	2.45	-2.40	14.81	0.28
SP0,400mM	SP400,200mM	4.89	2.45	-3.71	13.49	0.56
SP0,0mM	SP200,0mM	4.25	2.45	-4.35	12.85	0.72
SP200,200mM	SP400,200mM	4.14	2.45	-4.45	12.75	0.74
SP400,400mM	SP200,400mM	3.39	2.45	-5.21	11.99	0.89
SP400,200mM	SP0,200mM	2.58	2.45	-6.016	11.19	0.97

Level	- Level	Difference	Std Err Dif	Lower CL	Upper CL	p-Value
SP400,0mM	SP200,0mM	2.57	2.45	-6.03	11.17	0.97
SP0,0mM	SP400,0mM	1.67	2.45	-6.92	10.28	0.99
SP0,400mM	SP200,200mM	0.74	2.45	-7.86	9.34	1.00

Mineral nutrient concentrations in roots

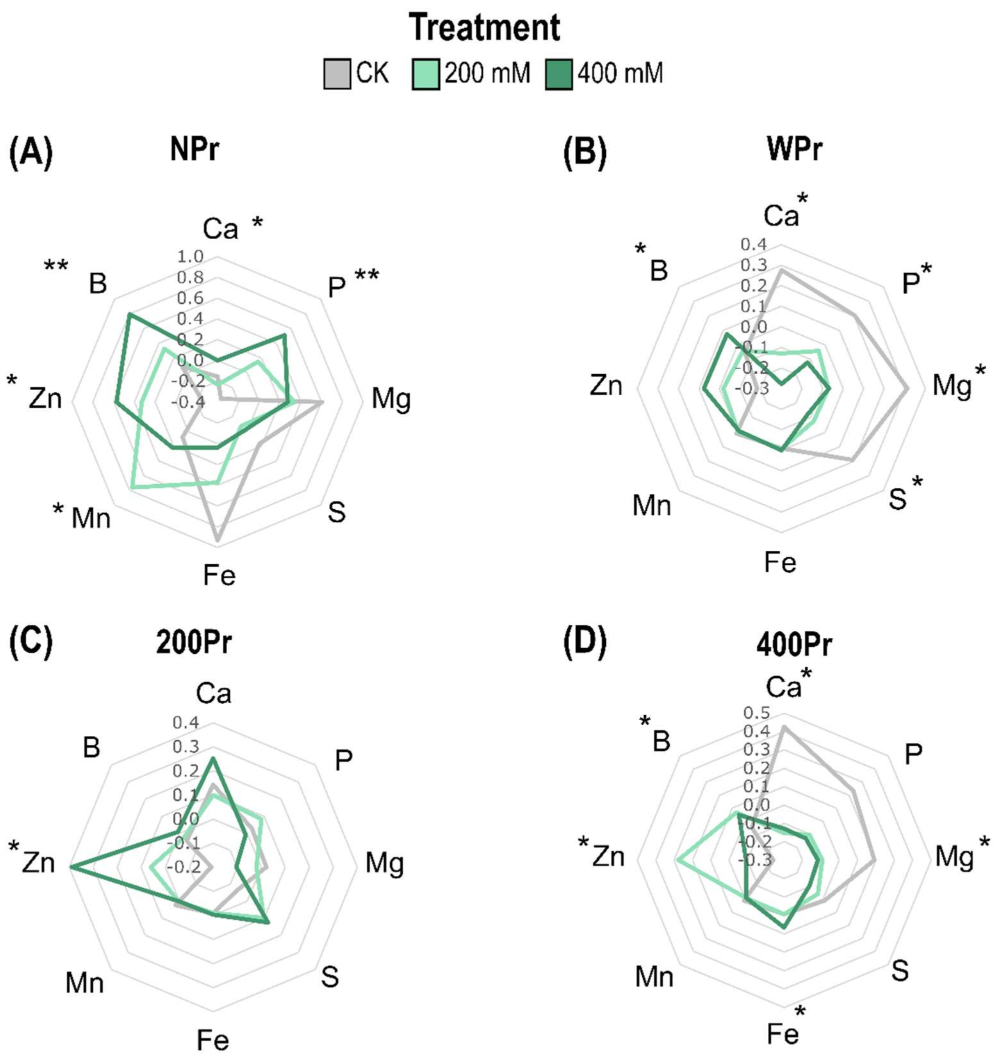

Figure S1: Radial plots showing root normalized mineral nutrient differences of *C. maritima* plants grown from non-primed (NPr) (A) and primed seeds (WPr (B), 200Pr (C), 400Pr (D)) under different NaCl treatments (CK, 200 mM, and 400 mM NaCl) for 7 days. Axes display Z-scores calculated per element. (n=6). One asterisk indicates significant differences in one treatment, and two asterisks in more than one treatment (Tukey test, $p < 0.05$).

Root Na^+ concentration

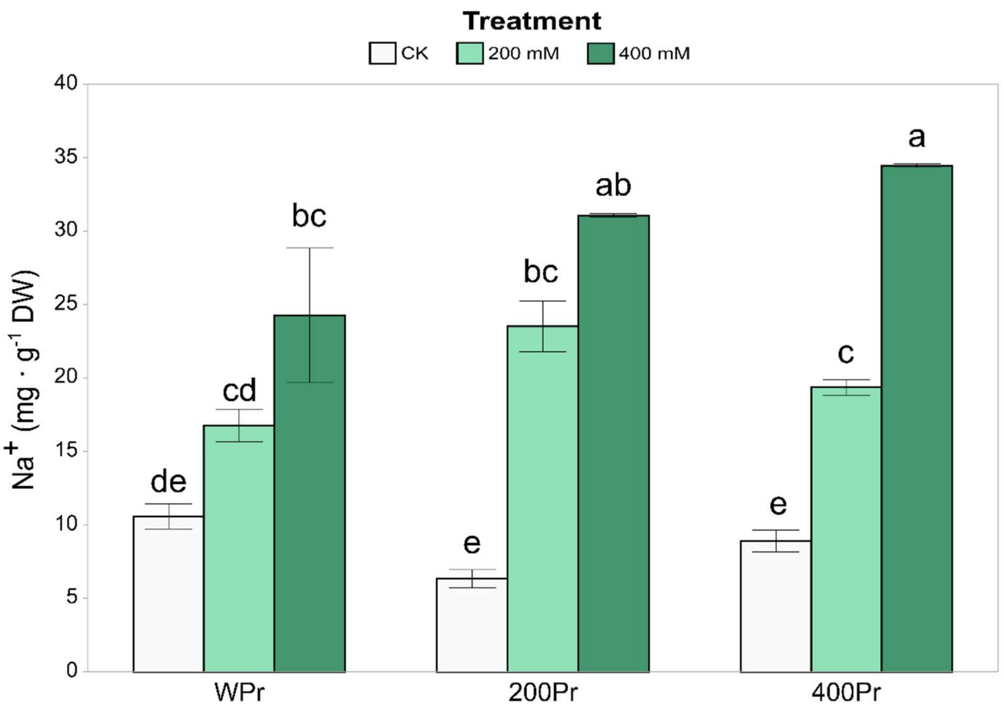

Figure S2: Root Na^+ concentration of *C. maritima* plants grown from primed seeds (WPr (B), 200Pr (C), 400Pr (D)) under different NaCl treatments (CK, 200 mM, and 400 mM NaCl) for 7 days. Values are presented as the mean ± SE (n = 6). Different letters indicate significant differences (Tukey test, $p < 0.05$).
